# Elevated plasma triglyceride concentration and risk of adverse clinical outcomes in 1.5 million people: a CALIBER linked electronic health record study

**DOI:** 10.1186/s12933-022-01525-5

**Published:** 2022-06-09

**Authors:** Riyaz S. Patel, Laura Pasea, Handrean Soran, Paul Downie, Richard Jones, Aroon D. Hingorani, Dermot Neely, Spiros Denaxas, Harry Hemingway

**Affiliations:** 1 Institute of Cardiovascular Sciences, University College London, 222 Euston Rd London, NW1 2DA UK; 2 London Biomedical Research Centre, NIHR University College, University College London and University College London Hospitals NHS Foundation Trust, London, UK; 3 UCL BHF Research Accelerator, UCL, London, UK; 4 Health Data Research UK London, University College London, London, UK; 5 Institute of Health Informatics, University College London, London, UK; 6 Department of Endocrinology, Diabetes and Metabolism, Manchester Royal Infirmary, Manchester, UK; 7 Department of Clinical Biochemistry, Bristol Royal Infirmary, Bristol, UK; 8 Global Medical Affairs, Akcea Therapeutics, Reading, UK; 9 Academic Health Science Network North East and North Cumbria (AHSN), Newcastle, UK; 10 The Alan Turing Institute, London, UK

**Keywords:** Triglycerides, Lipids, Pancreatitis, Diabetes, Myocardial infarction

## Abstract

**Background:**

Assessing the spectrum of disease risk associated with hypertriglyceridemia is needed to inform potential benefits from emerging triglyceride lowering treatments. We sought to examine the associations between a full range of plasma triglyceride concentration with five clinical outcomes.

**Methods:**

We used linked data from primary and secondary care for 15 M people, to explore the association between triglyceride concentration and risk of acute pancreatitis, chronic pancreatitis, new onset diabetes, myocardial infarction and all-cause mortality, over a median of 6–7 years follow up.

**Results:**

Triglyceride concentration was available for 1,530,411 individuals (mean age 56·6 ± 15·6 years, 51·4% female), with a median of 1·3 mmol/L (IQR: 0.9.to 1.9). Severe hypertriglyceridemia, defined as > 10 mmol/L, was identified in 3289 (0·21%) individuals including 620 with > 20 mmol/L. In multivariable analyses, a triglyceride concentration > 20 mmol/L was associated with very high risk for acute pancreatitis (Hazard ratio (HR) 13·55 (95% CI 9·15–20·06)); chronic pancreatitis (HR 25·19 (14·91–42·55)); and high risk for diabetes (HR 5·28 (4·51–6·18)) and all-cause mortality (HR 3·62 (2·82–4·65)) when compared to the reference category of ≤ 1·7 mmol/L. An association with myocardial infarction, however, was only observed for more moderate hypertriglyceridaemia between 1.7 and 10 mmol/L. We found a risk interaction with age, with higher risks for all outcomes including mortality among those ≤ 40 years compared to > 40 years.

**Conclusions:**

We highlight an exponential association between severe hypertriglyceridaemia and risk of incident acute and chronic pancreatitis, new diabetes, and mortality, especially at younger ages, but not for myocardial infarction for which only moderate hypertriglyceridemia conferred risk.

**Supplementary information:**

The online version contains supplementary material available at 10.1186/s12933-022-01525-5.

## Introduction

Despite being widely measured as part of routine lipid panels, the risks associated with severe hypertriglyceridemia remain incompletely understood. There has been a re-emergence of interest in understanding these risks for several reasons. First, the prevalence of hypertriglyceridemia is increasing worldwide as more individuals become obese and diabetics [1], the emergence of novel and costly therapies to lower triglyceride concentrations, will require better risk based prioritisation [2]. Third, the relationship between hypertriglyceridemia and coronary heart disease has been revisited with Mendelian Randomization studies suggesting a possible causal link [3].

Triglycerides in plasma are carried by chylomicrons and VLDL, collectively called triglyceride rich lipoproteins (TGRL). Hypertriglyceridemia ranges in severity from mild to very severe with differences in TGRL composition and metabolism [4]. Clinical guidelines broadly advocate lowering triglycerides when concentrations reach ~ 10 mmol/L, to reduce risk of acute pancreatitis, as a causal and mechanistic link is widely accepted [5, 6]. For myocardial infarction (MI), causality is far less certain, and measured triglyceride concentrations may represent more of a biomarker of risk, with risk mediated by smaller apoB carrying TGRL and remnants arising from incomplete lipolysis of larger TGRLs [7, 8]. In either case, there remains a need to fully understand the degree of risk associated with clinically measured hypertriglyceridemia, for relevant outcomes, especially at very high concentrations given these are now more prevalent, to aid clinical decision making and risk stratification.

In this regard, where observational data on hypertriglyceridemia in general populations exist, for different endpoints, including AP and MI studies have mostly examined people with mild to moderate triglyceride elevations between 1.7 and 10 mmol/L [9–14]. Higher ranges of triglycerides > 10 mmol/L have mostly been assessed in smaller studies of patients with prior pancreatitis, including those referred to lipid clinics, with associated selection bias [15]. In contrast, studies that have examined risks of severe hypertriglyceridemia in the general population, have been limited to evaluating only those with triglyceride concentrations > 5·6 mmol/L or > 11·3 mmol/L, often without reference to normal values or lacking external validity by using claims data or insurance databases [16, 17].

To provide a clearer understanding of the risks associated with hypertriglyceridemia, and especially severe hypertriglyceridemia in the general population, we report on the associations between a full spectrum of routinely measured triglyceride values from < 1·7 mmol/L to > 20 mmol/L and risks of multiple clinically relevant incident outcomes, including acute pancreatitis (AP), chronic pancreatitis (CP), myocardial infarction (MI), new onset diabetes and all-cause mortality. We do this using a representative population with linked primary care, secondary care and mortality records, leveraging the universal healthcare coverage provided by the NHS in England.

## Methods

### Study design and data sources

We conducted a cohort study (Additional file 2) using linked electronic health records (EHR) between primary care data (CPRD), hospital admissions (HES) and cause of death registry (ONS) in England from 1997 to 2016. CPRD has been shown to be representative of the English population by age, sex and ethnicity, and validated for research [18]. The study was approved by the MHRA (UK) Independent Scientific Advisory Committee 17_033, under Sect. 251 (NHS Social Care Act 2006). This study was carried out as part of the CALIBER resource (https://www.caliberresearch.org/**)** which provides validated EHR phenotyping algorithms and tools for national structured data sources [19].

### Study population

Patients of all ages were eligible for inclusion into the study if they had at least one triglyceride measurement recorded in CPRD and at least one year follow up. The baseline date for each patient was the date of their first triglyceride record that occurred at least 1 year after registration with their general practitioner (GP). They were followed up until they had an outcome, transferred out of their GP practice, died or until the administrative censoring date for data collection, up to a maximum of 5 years.

### Exposure

The primary exposure in this study, baseline triglyceride concentration, was categorised into the following five groups: ≤1·7 (reference, “normal”), > 1·7 − 4·5 (mild hypertriglyceridemia), > 4·5–10 (moderate hypertriglyceridemia), > 10–20 (severe hypertriglyceridemia), > 20 (very severe hypertriglyceridemia) mmol/L.

### Baseline covariate definitions

Baseline covariates included demographics (age and sex), behaviours (alcohol consumption and smoking status), prior medical history (AP, CP, diabetes, hypertension and MI), clinical biomarkers (body mass index (BMI), blood pressure and lipids [total cholesterol, low density lipoprotein cholesterol (LDL-C), high density lipoprotein cholesterol (HDL-C)]), and prescriptions for lipid regulating drugs (statins, fibrates, nicotinic acid, bile acid sequestrants and omega-3 fatty acids). Medical histories were defined as any record of a diagnosis prior to baseline date. Behaviours and clinical biomarkers were captured in primary care data and were taken as the most recent record in the year prior to a patient’s baseline date. Prescribed drugs were defined as any record of prescription in primary care prior to the baseline date.

### Follow up for clinical outcomes

Patients were followed-up for up for occurrences of new clinical outcome diagnoses made during the follow up period in primary care or hospital admissions records or listed as a cause of death in mortality records for: (1) acute pancreatitis, (2) chronic pancreatitis, (3) new-onset diabetes (type 1,2 or unspecified), (4) acute myocardial infarction and (5) all-cause mortality. The Read and ICD-10 codes used to define each of the covariates and outcomes can be found at the CALIBER portal.

### Statistical analyses

Patient characteristics grouped by baseline triglyceride concentration, were summarised using mean and standard deviation (SD) for continuous variables and frequency and percent for categorical variables. Kaplan-Meier plots were used to estimate the observed cumulative incidence of each outcome, stratified by triglyceride groups. For each outcome, Cox proportional hazard models were used to estimate hazard ratios and 95% confidence intervals for the risks across triglyceride groups with the reference category of ≤ 1·7 mmol/L. The proportional hazards assumption was checked using log (-log) and Schoenfeld residual plots. Each outcome was investigated independently and therefore patients could potentially have events contributing to all models. The presented models were:

1. Unadjusted for baseline covariates (univariable analysis).
2. Adjusted for age and sex.
3. Adjusted for age, sex, BMI, alcohol consumption, diabetes, smoking, hypertension, lipid lowering drug prescriptions, HDL, total cholesterol and stratified by general practice.

### Missing data

Covariate values missing at baseline were imputed using multiple imputation using chained equations (MICE). We imputed 10 datasets using models with all covariates and outcomes included to estimate missing values. In addition to analyses performed using multiply imputed data we fitted models using complete cases only. Calculated LDL-C data for triglyceride concentrations > 4.5 mmol/L were missing as LDL-C estimation is inaccurate at these levels when using the standard Friedewald equation and not usually reported clinically in these circumstances [20].

### Subgroup and sensitivity analyses

We conducted a-priori specified subgroup analyses: by age (≤ 40 years and > 40 years), choosing this cut point based on current guidelines for familial chylomicronaemia syndrome; history of prior pancreatitis (acute or chronic) and prescribed lipid lowering therapy. As some studies used peak triglyceride concentrations rather than first, we also fitted models using each patient’s peak triglyceride concentration recorded in a 12-month window prior to baseline date. Finally, we explored the shape of the association between baseline triglyceride and each outcome, by modelling continuous triglyceride concentrations using restricted cubic splines (3 knot points) in Cox proportional hazard models.

All analyses were performed using R (version 3·6).

## Results

In total, 1,530,441 patients were included in the study with a median follow-up time of 6·7 years (Interquartile range (IQR): 3·4–10·5 years). The median baseline triglyceride concentration was 1·3 mmol/L (IQR: 0·9 − 1·9). Patients were grouped according to their baseline triglyceride concentration: 1,053,783 (68·9%) had triglycerides ≤ 1·7 mmol/L, 443,768 (29·0%) had 1·7 − 4·5 mmol/L, 29,601 (1·9%) had 4·5-10 mmol/L, 2669 (0·2%) had 10-20 mmol/L, and 620 (0·04%) had > 20 mmol/L. In total 3289 (0·21%) patients had severe hypertriglyceridemia > 10 mmol/L. In follow up 980,302 (64%) individuals had at least one further triglyceride value recorded. Overall, individuals had a mean (SD) of 3.0 (4.2) triglyceride records in follow-up. 427,613 (27.9%) had at least one deviation from their baseline triglyceride category during follow-up, indicating reasonable stability of baseline triglyceride categories.

### Study population characteristics

The population characteristics reflected a clinical cohort from primary care and are described in Table 1. The population with a measured triglyceride concentration was relatively young (mean age 56·6 years (SD: 15·6 years), with a trend to higher concentrations among the younger age groups and those with prior AP. We also observed greater concentrations in those who were underweight (BMI < 18) as well as those who were obese (BMI > 30). There was an increased prevalence of both type 1 and 2 diabetes, more excess alcohol use and lower HDL as well as greater prescribing of lipid lowering drugs with increasing triglyceride concentrations (Table 1). Characteristics of the study population by age group (≤ 40 years and > 40 years) are presented in Additional file 1: Table S1.

**Table 1:** Patient baseline characteristics stratified by baseline triglycerides

	Baseline triglyceride (mmol/L)				
	≤ 1.7	(1.7,4.5]	(4.5,10]	(10,20]	> 20
	N = 1,053,783	N = 443,768	N = 29,601	N = 2669	N = 620
Age (years)	56.3 (16.2)	57.5 (14.2)	53.3 (12.8)	49.6 (11.5)	47.5 (11.0)
Women	583,257 (55.3)	193,656 (43.6)	8416 (28.4)	507 (19.0)	131 (21.1)
Behaviours					
Smoking status					
Non-Smoker	529,103 (50.2)	186,748 (42.1)	10,050 (34.0)	812 (30.4)	168 (27.1)
Ex-Smoker	298,989 (28.4)	137,105 (30.9)	9017 (30.5)	785 (29.4)	177 (28.5)
Smoker	153,692 (14.6)	82,859 (18.7)	7488 (25.3)	808 (30.3)	202 (32.6)
Missing %	6.8	8.4	10.3	9.9	11.8
Alcohol consumption					
Non-Drinker	142,455 (13.5)	61,074 (13.8)	3789 (12.8)	297 (11.1)	67 (10.8)
Ex-Drinker	14,672 (1.4)	7060 (1.6)	567 (1.9)	71 (2.7)	12 (1.9)
Occasional Drinker	189,678 (18.0)	79,067 (17.8)	4480 (15.1)	339 (12.7)	82 (13.2)
Current Drinker	472,984 (44.9)	192,133 (43.3)	12,275 (41.5)	1061 (39.8)	230 (37.1)
Excess Drinker	80,606 (7.6)	41,810 (9.4)	4255 (14.4)	499 (18.7)	127 (20.5)
Missing %	14.6	14.1	14.3	15.1	16.5
Medical history					
Acute Pancreatitis	4816 (0.5)	2865 (0.6)	319 (1.1)	65 (2.4)	28 (4.5)
Chronic pancreatitis	1296 (0.1)	692 (0.2)	103 (0.3)	23 (0.9)	6 (1.0)
Diabetes					
Unspecified type	2189 (0.2)	1277 (0.3)	192 (0.6)	20 (0.7)	5 (0.8)
Type 1	9265 (0.9)	2415 (0.5)	234 (0.8)	36 (1.3)	11 (1.8)
Type 2	50,757 (4.8)	40,641 (9.2)	4113 (13.9)	485 (18.2)	117 (18.9)
Hypertension	273,952 (26.0)	150,843 (34.0)	9733 (32.9)	734 (27.5)	155 (25.0)
Myocardial infarction	40,361 (3.8)	20,898 (4.7)	1367 (4.6)	97 (3.6)	13 (2.1)
Atrial fibrillation	26,349 (2.5)	10,662 (2.4)	619 (2.1)	49 (1.8)	14 (2.3)
Heart failure	24,073 (2.3)	11,783 (2.7)	803 (2.7)	62 (2.3)	14 (2.3)
Ischaemic stroke	7729 (0.7)	3053 (0.7)	159 (0.5)	8 (0.3)	< 5
Clinical biomarkers					
BMI	27.6 (5.93)	30.3 (5.89)	31.0 (5.46)	31.2 (5.69)	30.3 (5.58)
Underweight	9645 (0.9)	913 (0.2)	38 (0.1)	6 (0.2)	< 5
Normal Weight	149,598 (14.2)	30,583 (6.9)	1389 (4.7)	112 (4.2)	33 (5.3)
Overweight	154,601 (14.7)	74,418 (16.8)	5008 (16.9)	420 (15.7)	90 (14.5)
Obese	117,547 (11.2)	87,663 (19.8)	6901 (23.3)	610 (22.9)	124 (20.0)
Missing %	59.1	56.4	54.9	57	59.5
SBP (mmHg)	137 (20.6)	142 (20.1)	142 (20.0)	142 (19.2)	141 (20.1)
Missing %	22.8	19.6	21.9	26.2	31.3
DBP (mmHg)	80.9 (11.5)	83.6 (11.4)	85.2 (11.7)	86.3 (11.7)	86.4 (11.4)
Missing %	22.8	19.6	21.9	26.2	31.3
HDL (mmol/L), Median (IQR)	1.500 (1.20, 1.8)	1.200 (1.00, 1.4)	1.000 (0.90, 1.2)	0.900 (0.75, 1.1)	0.995 (0.70, 1.4)
Missing %	10.6	13.1	17.9	28.7	42.9
LDL (mmol/L), Median (IQR)	3.10 (2.50, 3.80)	3.50 (2.80, 4.20)	NA	NA	NA
Missing %	17.3	22.5	–	–	–
Total cholesterol (mmol/L), Median (IQR)	5.2 (4.5, 5.9)	5.8 (5.1, 6.6)	6.4 (5.6, 7.4)	7.6 (6.6, 8.8)	10.2 (8.2, 12.7)
Missing %	2	1.9	2.4	4	7.1
Prescribed drugs					
Statins	150,926 (14.3)	84,022 (18.9)	6107 (20.6)	586 (22.0)	160 (25.8)
Fibrates	7196 (0.7)	7283 (1.6)	1181 (4)	178 (6.7)	53 (8.5)
Statin + fibrates	965 (0.1)	1412 (0.3)	339 (1.1)	59 (2.2)	19 (3.1)
Nicotinic acid	251 (0.0)	239 (0.1)	42 (0.1)	10 (0.4)	< 5
Omega-3 fatty acids	1523 (0.1)	1001 (0.2)	172 (0.6)	34 (1.3)	10 (1.6)
Other lipid lowering drugs	6871 (0.7)	4616 (1.0)	420 (1.4)	44 (1.6)	11 (1.8)
Follow up time, Median (IQR)	6.40 (3.26, 10.2)	7.21 (3.69, 11.1)	7.41 (3.71, 11.6)	7.26 (3.75, 11.3)	7.14 (3.63, 11.4)

Presented numbers are mean (standard deviation) for continuous variables or frequency (%) for categorical variables, unless otherwise stated

### Association between baseline triglyceride concentrations and clinical outcomes

**Fig. 1:**
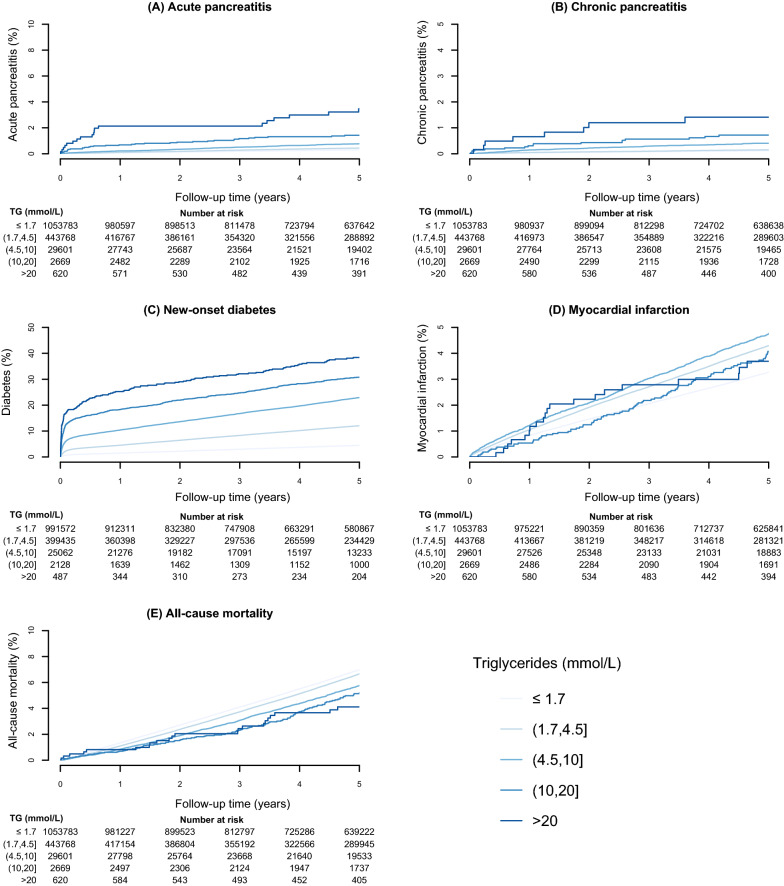
Kaplan-Meier analysis for triglyceride groups and clinical outcomes. Absolute risk at each triglyceride strata for each of the 5 clinical outcomes over 5 years is presented, along with numbers at risk

**Fig. 2:**
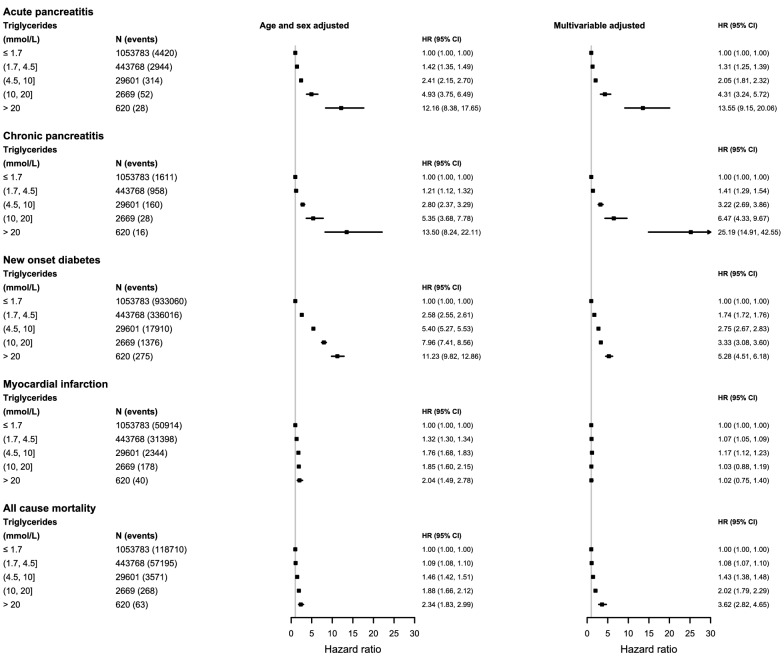
Forest plots of hazard ratios for association of triglyceride concentrations and clinical outcomes. Models include age and sex adjusted and fully multivariable adjusted for age, sex BMI, alcohol consumption, diabetes, smoking, hypertension, lipid lowering drug prescriptions, HDL, total cholesterol and stratified by general practice

Over a median follow up of 6·7 years, there were 7758 (0·5%) AP admissions; 2773 (0·1%) CP diagnoses; 143,209 (9·4%) new onset diabetes diagnoses; 84,874 (5·5%) MI admissions along with 179,807 (11·7%) deaths among the cohort. Figures 1 and 2 illustrate the associations between triglyceride concentrations and each outcome.

Compared to the referent group with a triglyceride concentration ≤ 1·7 mmol/L we found the following absolute and relative risks at five years:

#### Acute pancreatitis

The risk for AP was highest in the > 20 mmol/L group (3·5%, 95% CI: 1·9, 5·0) compared to the referent group (0·3% (95% CI: 0·2, 0·3), (Fig. 1A). After multivariable adjustment, equating to a 13-fold increase in relative risk (HR: 13·55 (9·15, 20·06)) (Fig. 2).

#### Chronic pancreatitis

The risk for those with concentrations > 20 mmol/L was 1·4% (95% CI: 0·4, 2·4) compared to 0·12% (95% CI: 0·11, 0·13)) in the referent group (Fig. 1B), and a 25-fold increase in relative risk (HR: 25·19 (14·91, 42·55) (Fig. 2).

#### Diabetes mellitus

The risk of developing new onset diabetes (any type) was highest in the > 20 mmol/L group, with 38% of the population developing the disease (95% CI: 33·7, 42·8), compared with 4·5% (95% CI: 4·4, 4·5) in the referent group. (Fig. 1C). Adjusted for covariates there remained a 5-fold increase in relative risk (HR: 5·28 (4·51, 6·18) (Fig. 2).

#### Myocardial infarction

Unlike other outcomes, the risk of MI was highest in the moderate triglyceride group (4·5-10 mmol/L (4·8%)), not the highest triglyceride group (> 20 mmol/L (3·7%)) (Fig. 1D). After adjustment, only those with mild and moderate triglyceride elevation (1·7 –4·5 and 4·5–10 mmol/L) had a modest but statistically significant risk of incident MI compared to the referent group (HR: 1·07 (1·05, 1·09) and 1·17 (1·12, 1·23) respectively) (Fig. 2).

#### All-cause mortality

The risk of death was 4·1% (95% CI: 2·4, 5·8) in those with triglyceride concentrations > 20 mmol/L compared to 7·0% (95% CI: 6·9, 7·0) in the referent group (Fig. 1E). However, in adjusted analysis, accounting for the confounding effect of age and other risk factors, we observed a near fourfold increased relative risk for those with triglyceride concentrations > 20 mmol/L compared to the referent group (HR: 3·62 (2·82, 4·65)) (Fig. 2).

The hazard ratios for all variables in the multivariable models described are presented in Additional file 1: Table S2. In addition to triglycerides, we observed associations between alcohol consumption, smoking status, diabetes and hypertension and the outcomes.

### Subgroup analyses

#### Age group

**Fig. 3:**
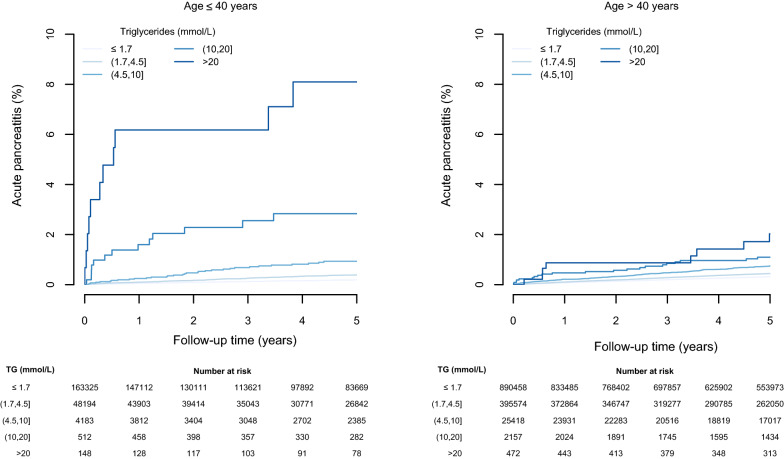
Kaplan-Meier analysis for triglyceride groups and AP stratified by age group ≤ 40 years and > 40 years

Risks from severe hypertriglyceridemia were greater in those ≤ 40 years compared to those > 40 years (Fig. 3, Additional file 1: Table S3). In younger people, with triglyceride concentrations of > 20 mmol/L, there was a near 40-fold increase in risk of AP compared to the referent group (HR 37·3 (20·9, 66·5)), versus a comparable 8-fold risk in those aged > 40 years (HR 8·5 (5·2, 13·9)) (Additional file 1: Figure S2). Similar estimates were observed for CP (Additional file 1: Figures S1, S2; Table S3). The risk of diabetes at triglyceride concentrations > 20 mmol/L compared to < 1.7 mmol/L was twice as high in those ≤ 40 years compared to those > 40 years (Additional file 1: Figure S2). Event rates for MI and all-cause mortality were higher in the older age group across all triglyceride groups. (Additional file 1: Figure S1; Table S3), but the relative association of MI with moderate triglyceride concentrations was also greater in those aged ≤ 40 years (Additional file 1: Figure S2).

#### Prior pancreatitis

The absolute risks of AP at 5 years in patients with prior pancreatitis ranged from 9·4–37.2% in those with triglyceride concentrations ≤ 1·7 mmol/L and > 20 mmol/L, respectively. Corresponding risks in those without prior pancreatitis were 0·3% and 1·8% (Additional file 1: Figure S3; Table S4). Similarly, higher risks of CP, diabetes, MI and all-cause mortality were also observed in patients with prior pancreatitis. Despite the higher incidence of events, the relative risk association between triglyceride concentrations and all clinical outcomes was attenuated when compared to those without prior pancreatitis. (Additional file 1: Figure S4).

#### Prescribed lipid lowering therapy

**Fig. 4:**
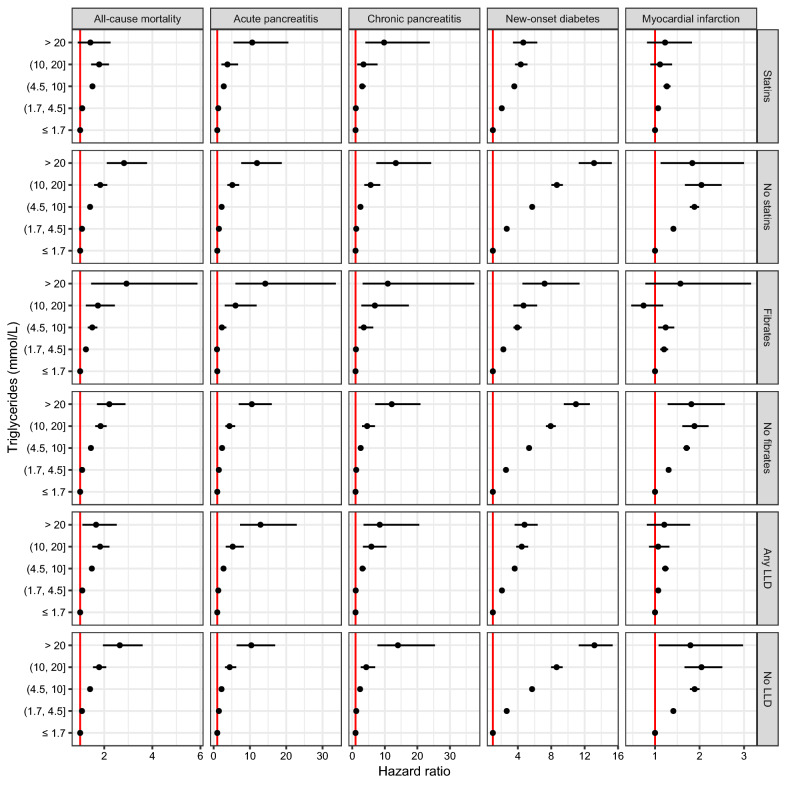
Hazard ratios for triglyceride concentrations and clinical outcomes, adjusted for age and sex and stratified by lipid lowering therapy prescriptions [statins (n = 241,801), no statins (n = 1,288,640), fibrates(n = 15,891), no fibrates (n = 1,514,550), any LLD (n = 250,806), no LLD’s (n = 1,279,635)]

Overall, risk associations were attenuated among people taking statins or any lipid lowering therapy, including fibrates, for risk of MI and new onset diabetes, and to a lesser extent for all-cause mortality. However, for both AP and CP, there was little difference in risk for patients with 10-20 mmol/L and > 20 mmol/L of triglycerides whether on prior lipid lowering treatment or not. Figure 4.

### Sensitivity analysis

We also explored the association with outcomes using continuous triglyceride concentrations. Age-sex adjusted associations using restricted cubic splines (3 knot points) is provided in supplementary materials. Notably, increasing triglyceride concentrations were associated exponentially with increasing risk for each outcome (Additional file 1: Figure S5).

We repeated our analyses using the highest recorded triglyceride within a 12-month window as opposed to the first recorded concentration. The median peak triglyceride concentration across patients was 1·63 mmol/L (IQR: 0·91 –1·94). Overall, association estimates for each clinical outcome were similar to the primary analysis (Additional file 1: Table S5).

Finally, to ensure our imputation approach did not bias our results, we repeated analyses using non-missing data, and found similar estimates for association with each of the 5 clinical outcomes and triglyceride concentrations (Additional file 1: Table S6).

## Discussion

Using nationally linked primary and secondary care electronic health records in over 1·5 M people with a triglyceride measurement, we report on the associations between a full range of triglyceride concentrations from < 1·7 to > 20 mmol/L and multiple clinically relevant outcomes. We observed four novel findings: (1) the risk of MI is modest and observed with moderately elevated triglyceride concentration but not severe hypertriglyceridemia; (2) all observed risks were greater in younger people where primary rather than secondary causes of hypertriglyceridemia are likely to predominate, (3) lipid lowering medication prescribing at baseline did not attenuate the risk between high triglycerides and incident acute or chronic pancreatitis and (4) the observed risks from raised triglyceride concentration are continuous and exponential starting at values much lower than established treatment thresholds. Our findings inform our evolving understanding of the full spectrum of risks associated with hypertriglyceridemia.

Prior studies in general population settings, have mostly explored risk associated with moderate elevations in triglyceride concentration, [9–14] or sought to examine only the higher range of triglycerides. For example, Toth et al. found those with a triglyceride concentration > 22·8 mmol/L had a 12-fold increase in risk for AP compared to those < 8·6 mmol/L [16].  A more recent Israeli study, again from a health insurance provider, found a stepwise increase in risk for severe hypertriglyceridemia with AP but not with CVD [17]. However, both studies had limited external validity coming from insurance databases and only explored two outcomes, without reference to more usual triglyceride concentrations. Our study adds to the existing literature, by reporting simultaneously on 5 relevant clinical outcomes across the full range of triglycerides encountered in a representative primary care population.

The incidence of AP in our study was low, but consistent with prior studies [15, 21]. Pederson et al. reported an 11-fold risk for AP for those with concentrations > 5 mmol/L, compared to the lowest group < 1.0  mol/L, although the lower than usual referent group concentration could have inflated the risk estimate. Importantly with only 2100 individuals having triglycerides > 5·0 mmol/L, information for severe hypertriglyceridemia was limited [9]. We extend this work, including 3300 patients with hypertriglyceridemia > 10 mmol/L, finding a 13-fold increase in future risk of AP for those with triglyceride > 20 mmol/L compared to those with more usual concentrations of ≤-1.7 mmol/L. We also found that young patients had significantly higher risk from severe hypertriglyceridemia pointing to potentially different aetiologies with genetic factors contributing to risk earlier in life [22]. For example, prevalence of FCS is thought to be enriched in this group and diagnostic criteria include an AP event at ≤ 40 years [23]. Finally in multivariable analysis we noted that alcohol excess and smoking were independent predictors of AP, highlighting the important role of lifestyle factors in this condition [24].

We found that CP risk followed AP risk with even higher estimates, perhaps unsurprising as these are not always mutually exclusive [25]. Diabetes is usually reported as a cause of hypertriglyceridemia rather than a consequence [26]. Yet here we demonstrate that high triglyceride concentrations may be a quantifiable precursor to diabetes, both from insulin insufficiency and resistance with a remarkably linear risk. Triglyceride concentrations are known to be associated with insulin resistance and are a sensitive and early marker of metabolic syndrome [27]. Of note, in our analysis 11% of the new onset diabetes diagnoses occurred within 30 days of the baseline triglyceride measurement. This is likely explained by blood testing identifying both high triglycerides and high glycated haemoglobin (HbA1c) and a recording of diabetes in the electronic health record in the days afterwards. The associated cardiovascular risk from metabolic dyslipidaemia along with greater AP risk for those in the highest triglyceride strata may together explain the association with mortality, which has also been reported previously in a follow up study of the Bezafibrate Infarction Prevention (BIP) trial [28].

Of particular interest is the association between triglycerides and MI. Observational studies have repeatedly demonstrated that mild-to-moderate hypertriglyceridemia is associated with a higher risk of MI, but when adjusted for non-HDL-C the risk is usually attenuated [12]. These findings are consistent with the idea that cardiovascular risk associated with hypertriglyceridemia is mediated by atherogenic apoB containing TGRLs and remnants, captured by non-HDL, and not the triglycerides per se [7]. Our findings, extend prior work by including the full range of triglyceride concentrations including severe hypertriglyceridemia, and provide similar results. The lack of association with severe hypertriglyceridemia is likely explained by the greater dominance of large triglycerides containing lipoprotein particles (mainly chylomicrons and large VLDLs) at these levels, which themselves are too large to enter the intima and thus less atherogenic than smaller lipoprotein particles [29]. In contrast more moderate hypertriglyceridemia may be a marker for greater burden of smaller atherogenic TGRL. Of note, patients ≤ 40 years with moderate hypertriglyceridemia had the highest risk for MI, potentially due to additional contributions from inherited mixed dyslipidaemias such as Familial Combined Hyperlipidaemia and more atherogenic lipoproteins conferring atherosclerotic risk [30]. Of course, MI risk could also be explained by the co-presence of other metabolic disturbances such as insulin resistance, diabetes and obesity themselves contributing to triglyceride concentrations in this range [7, 31].

Statin use in our study was associated with a lower risk for MI and death for any given concentration of triglycerides, likely due to the LDL-C lowering effect and associated CVD risk benefit [32]. The lower risk of new onset diabetes among statin users is unusual given the small statin mediated increase in the risk of new onset diabetes in major clinical trials, although these typically excluded those with fasting triglycerides > 4·5 mmol/L. Our finding could reflect an unmeasured bias such as greater lifestyle and medical intervention efforts to prevent diabetes in those attending GP visits and prescribed statins compared to those who were not. Among patients taking fibrates, risks for all clinical events were only marginally lower compared to those not on fibrates which is perhaps unsurprising given some studies have found an increase in risk of pancreatitis with fibrate use[33, 34]. Importantly, lipid lowering therapy of any kind was not associated with reduced risk for AP and CP at triglyceride concentrations > 10 or > 20 mmol/L indicating perhaps a ceiling of effect for commonly used lipid lowering therapies, and highlighting the need for novel triglyceride lowering agents [35].

Our study has several limitations. First, as is common with real world data, fasting status was unclear within clinical records. However, our risk estimates for moderate hypertriglyceridemia and AP were similar to published cohort studies with fasting samples and there is recognition that non-fasting samples may be more indicative of health risk[9, 11]. Second, we had significant missing data for relevant comorbidities, which we resolved by using imputation techniques. Sensitivity analyses did not indicate any major distortion of estimates with complete case analysis. Third, we used the first recorded triglyceride measurement rather than peak, given that in clinical practice clinicians rarely wait to determine the highest reading but intervene with the data to hand. Sensitivity analyses did not show any meaningful difference in associations when peak or first values were used. Finally, while we did not include any events on the same day as the baseline triglyceride measure, we did not censor events occurring within 30 days, and therefore some events may have co-occurred if there was a delay in recording. However, aside from diabetes (explained above), the separate data sources for hospital admissions (secondary care) and blood tests (primary care) limits this possibility as does the low rate of the non-diabetes outcomes occurring within 30 days (0.6 to 3%).

Clinically, our findings provide insight into the serious health risks associated with both moderate and severe hypertriglyceridemia, especially in younger patients. Our continuous trait analysis identifies that the risk of adverse outcomes, especially for acute pancreatitis increases exponentially once triglycerides are just moderately elevated. This finding potentially challenges current treatment thresholds, [5, 6] while supporting guidelines advocating intervention at lower triglyceride concentrations[36]. Further, our findings support measurement of triglycerides for cardiovascular risk management, if only as a marker of TGRLs, for which emerging therapies may provide cardiovascular benefit [37]. Finally, unlike many studies on triglycerides our findings represent “real world” association analyses, with patients who have treated and untreated lipids and various comorbidities. As such the findings are more generalizable but could also explain why some estimates may differ from more bespoke studies.

In conclusion, we show in over 1·5 M people that moderate and severe triglyceride elevation is associated with risk of AP and CP, new onset diabetes, and death, but for MI only moderate hypertriglyceridemia is associated with risk. Of note, these risks are continuous and start at modest elevations of triglyceride concentrations, are higher in younger people and current drugs may have limited impact for reducing these risks from severe and very severe hypertriglyceridemia. We anticipate these findings will support clinical interpretation of triglyceride concentrations, decisions for investigation and treatment, and cost effectiveness analyses for emerging triglyceride lowering therapies.

## Supplementary Information

**Additional file 1**. STROBE Statement—checklist of items that should be included in reports of observational studies.

**Additional file 2: Table S1**. Baseline characteristics of the study population by age group. **Table S2**. Hazard ratios for all variables in the multivariable models. **Table S3**. Kaplan Meier point estimates (%) at 1 and 5 years for each clinical outcome stratified by baseline TG and age >40 and ≤40 years. **Table S4**. Kaplan Meier point estimates at 1 and 5 years for each clinical outcome stratified by baseline TG and prior pancreatitis. **Table S5**. Hazard ratios and 95% confidence intervals from multivariable cox regression models using patients peak triglyceride record over 12 months. **Table S6**. Hazard ratios and 95% confidence intervals from multivariable cox regression models using complete-cases only (complete-cases n=505,579 overall and n= 446,091 for the subset of diabetes-free patients). **Figure S1**. Kaplan Meier curves for association of triglycerides and clinical outcomes stratified by age >40 and ≤40 years. **Figure S2**. Hazard ratios for triglyceride values and clinical outcomes, adjusted for age and sex and stratified by Age ≤ 40 or > 40 years. **Figure S3**. Kaplan Meier curves for association of triglycerides and clinical outcomes stratified by prior pancreatitis. **Figure S4**. Hazard ratios for triglyceride values and clinical outcomes, adjusted for age and sex and stratified by prior pancreatitis. **Figure S5**. Multivariable adjusted association between triglycerides and each endpoint. Triglyceride level was fitted in the model using restricted cubic splines (3 knot points).

## Data Availability

The data that support the findings of this study are available from the Clinical Practice Research Datalink (https://www.cprd.com/) but restrictions apply to the availability of these data, which were used under license for the current study, and so are not publicly available. Due to privacy laws and the data user agreement between the University College London and Clinical Practice Research Datalink, authors are not authorised to share individual patient data from these electronic health records. Requests to access data provided by Clinical Practice Research Datalink (CPRD) should be sent to CPRD. The CALIBER phenotype Library (https://www.caliberresearch.org/portal/) offers open sharing of phenotypic and analytic algorithms for use by other researchers/.
