# Supplementary material for: Elevated plasma triglyceride concentration and risk of adverse clinical outcomes in 1.5 million people: a CALIBER linked electronic health record study
Source: Cardiovasc Diabetol. 2022 Jun 9;21:102. doi: 10.1186/s12933-022-01525-5 (PMC9185961; doi:10.1186/s12933-022-01525-5)
Supplement: Supplementary file 1 — Additional file 1. STROBE Statement—checklist of items that should be included in reports of observational studies. [file 12933_2022_1525_MOESM1_ESM.docx]

STROBE Statement—checklist of items that should be included in reports of observational studies

|  | Item No. | Recommendation | Page  No. | Relevant text |
| --- | --- | --- | --- | --- |
| **Title and abstract** | 1 | (*a*) Indicate the study’s design with a commonly used term in the title or the abstract | 1 | Title |
|  |  | (*b*) Provide in the abstract an informative and balanced summary of what was done and what was found | 2 | Abstract |
| Introduction | | | |  |
| Background/rationale | 2 | Explain the scientific background and rationale for the investigation being reported | 3 | Introduction (paragraphs 1-3) |
| Objectives | 3 | State specific objectives, including any prespecified hypotheses | 3 | Introduction (paragraph 4) |
| Methods | | | |  |
| Study design | 4 | Present key elements of study design early in the paper | 4 | Methods (paragraph 1) |
| Setting | 5 | Describe the setting, locations, and relevant dates, including periods of recruitment, exposure, follow-up, and data collection | 4 | Methods (paragraphs 1-5) |
| Participants | 6 | Give the eligibility criteria, and the sources and methods of selection of participants. Describe methods of follow-up | 4 | Methods (paragraphs 2, 5) |
| Variables | 7 | Clearly define all outcomes, exposures, predictors, potential confounders, and effect modifiers. Give diagnostic criteria, if applicable | 4-5 | Methods (paragraphs 4,5) |
| Data sources/ measurement | 8* | For each variable of interest, give sources of data and details of methods of assessment (measurement). Describe comparability of assessment methods if there is more than one group | 4-5 | Methods (paragraphs 4,5) |
| Bias | 9 | Describe any efforts to address potential sources of bias | 5 | Methods (paragraphs 6,7,8) |
| Study size | 10 | Explain how the study size was arrived at | - | - |

Continued on next page

| Quantitative variables | 11 | Explain how quantitative variables were handled in the analyses. If applicable, describe which groupings were chosen and why | - | - |
| --- | --- | --- | --- | --- |
| Statistical methods | 12 | (*a*) Describe all statistical methods, including those used to control for confounding | 5 | Methods (paragraph 6) |
|  |  | (*b*) Describe any methods used to examine subgroups and interactions | 5 | Methods (paragraph 8) |
|  |  | (*c*) Explain how missing data were addressed | 5 | Methods (paragraph 7) |
|  |  | (*d*) If applicable, explain how loss to follow-up was addressed | 4-5 | Methods (paragraph 2,5) |
|  |  | (*e*) Describe any sensitivity analyses | 5 | Methods (paragraph 8) |
| Results | | | | |
| Participants | 13* | (a) Report numbers of individuals at each stage of study—eg numbers potentially eligible, examined for eligibility, confirmed eligible, included in the study, completing follow-up, and analysed | 6 | Results (paragraph 1) |
|  |  | (b) Give reasons for non-participation at each stage | n/a |  |
|  |  | (c) Consider use of a flow diagram | n/a |  |
| Descriptive data | 14* | (a) Give characteristics of study participants (eg demographic, clinical, social) and information on exposures and potential confounders | 6 | Results (paragraph 2) Table 1 |
|  |  | (b) Indicate number of participants with missing data for each variable of interest | Table 1 |  |
|  |  | (c) Summarise follow-up time (eg, average and total amount) | 6 | Results (paragraph 1) |
| Outcome data | 15* | Report numbers of outcome events or summary measures over time | 6 | Results (paragraph 3) |
| Main results | 16 | (*a*) Give unadjusted estimates and, if applicable, confounder-adjusted estimates and their precision (eg, 95% confidence interval). Make clear which confounders were adjusted for and why they were included | 6  Figure 1  Figure 2 | Results (paragraph 3) |
|  |  | (*b*) Report category boundaries when continuous variables were categorized | - | - |
|  |  | (*c*) If relevant, consider translating estimates of relative risk into absolute risk for a meaningful time period | - | - |

Continued on next page

| Other analyses | 17 | Report other analyses done—eg analyses of subgroups and interactions, and sensitivity analyses | 7-8 | Results (paragraphs 4-9) |
| --- | --- | --- | --- | --- |
| Discussion | | | | |
| Key results | 18 | Summarise key results with reference to study objectives | 8-9 | Discussion (paragraphs 1-6) |
| Limitations | 19 | Discuss limitations of the study, taking into account sources of potential bias or imprecision. Discuss both direction and magnitude of any potential bias | 10 | Discussion (paragraph 7) |
| Interpretation | 20 | Give a cautious overall interpretation of results considering objectives, limitations, multiplicity of analyses, results from similar studies, and other relevant evidence | 10-11 | Discussion (paragraphs 8,9) |
| Generalisability | 21 | Discuss the generalisability (external validity) of the study results | 11 | Discussion (paragraph 9) |
| Other information | |  | | |
| Funding | 22 | Give the source of funding and the role of the funders for the present study and, if applicable, for the original study on which the present article is based | 11 |  |
