# Supplementary material for: Elevated plasma triglyceride concentration and risk of adverse clinical outcomes in 1.5 million people: a CALIBER linked electronic health record study
Source: Cardiovasc Diabetol. 2022 Jun 9;21:102. doi: 10.1186/s12933-022-01525-5 (PMC9185961; doi:10.1186/s12933-022-01525-5)
Supplement: Supplementary file 2 — Additional file 2: Table S1. Baseline characteristics of the study population by age group. Table S2. Hazard ratios for all variables in the multivariable models. Table S3. Kaplan Meier point estimates (%) at 1 and 5 years for each clinical outcome stratified by baseline TG and age >40 and ≤40 years. Table S4. Kaplan Meier point estimates at 1 and 5 years for each clinical outcome stratified by baseline TG and prior pancreatitis. Table S5. Hazard ratios and 95% confidence intervals from multivariable cox regression models using patients peak triglyceride record over 12 months. Table S6. Hazard ratios and 95% confidence intervals from multivariable cox regression models using complete-cases only (complete-cases n=505,579 overall and n= 446,091 for the subset of diabetes-free patients). Figure S1. Kaplan Meier curves for association of triglycerides and clinical outcomes stratified by age >40 and ≤40 years. Figure S2. Hazard ratios for triglyceride values and clinical outcomes, adjusted for age and sex and stratified by Age ≤ 40 or > 40 years. Figure S3. Kaplan Meier curves for association of triglycerides and clinical outcomes stratified by prior pancreatitis. Figure S4. Hazard ratios for triglyceride values and clinical outcomes, adjusted for age and sex and stratified by prior pancreatitis. Figure S5. Multivariable adjusted association between triglycerides and each endpoint. Triglyceride level was fitted in the model using restricted cubic splines (3 knot points). [file 12933_2022_1525_MOESM2_ESM.docx]

**Supplementary Materials**

Patel et al. Elevated plasma triglyceride concentration and risk of adverse clinical events in 1.5 million people: A CALIBER linked electronic health record study

**List of supplementary tables and figures**

| Supplementary Table 1 | Baseline characteristics of the study population by age group |
| --- | --- |
| Supplementary Table 2 | Hazard ratios for all variables in the multivariable models |
| Supplementary Figure 1 | Kaplan Meier curves for association of triglycerides and clinical outcomes stratified by age >40 and ≤40 years |
| Supplementary Table 3 | Kaplan Meier point estimates (%) at 1 and 5 years for each clinical outcome stratified by baseline TG and age >40 and ≤40 years |
| Supplementary Figure 2 | Hazard ratios for triglyceride values and clinical outcomes, adjusted for age and sex and stratified by Age ≤ 40 or > 40 years |
| Supplementary Figure 3 | Kaplan Meier curves for association of triglycerides and clinical outcomes stratified by prior pancreatitis |
| Supplementary Table 4 | Kaplan Meier point estimates at 1 and 5 years for each clinical outcome stratified by baseline TG and prior pancreatitis |
| Supplementary Figure 4 | Hazard ratios for triglyceride values and clinical outcomes, adjusted for age and sex and stratified by prior pancreatitis |
| Supplementary Figure 5 | Multivariable adjusted association between triglycerides and each endpoint. Triglyceride level was fitted in the model using restricted cubic splines (3 knot points) |
| Supplementary Table 5 | Hazard ratios and 95% confidence intervals from multivariable cox regression models using patients peak triglyceride record over 12 months |
| Supplementary Table 6 | Hazard ratios and 95% confidence intervals from multivariable cox regression models using complete-cases only (complete-cases n=505,579 overall and n= 446,091 for the subset of diabetes-free patients) |

**Supplementary table 1: Baseline characteristics of the study population by age group**

|  | **Age ≤40 years** | **Age >40 years** |
| --- | --- | --- |
|  | **N=216362** | **N=1314079** |
| Age (years) | 31.8 ( 6.71) | 60.6 (12.61) |
| Women, n (%) | 111013 (51.3) | 674954 (51.4) |
| Smoking status, n (%) |  |  |
| Non-Smoker | 105293 (48.7) | 621588 (47.3) |
| Ex-Smoker | 43462 (20.1) | 402611 (30.6) |
| Smoker | 47696 (22.0) | 197353 (15.0) |
| Missing % | 9.2 | 7.0 |
| Alcohol consumption, n (%) |  |  |
| Non-Drinker | 34322 (15.9) | 173360 (13.2) |
| Ex-Drinker | 2979 (1.4) | 19403 (1.5) |
| Occasional Drinker | 33422 (15.4) | 240224 (18.3) |
| Current Drinker | 81332 (37.6) | 597351 (45.5) |
| Excess Drinker | 14468 (6.7) | 112829 (8.6) |
| Missing % | 23.0 | 13.0 |
| Acute pancreatitis, n (%) | 735 (0.3) | 7358 (0.6) |
| Chronic pancreatitis, n (%) | 227 (0.1) | 1893 (0.1) |
| Diabetes type, n (%) |  |  |
| Unspecified | 508 (0.2) | 3175 (0.2) |
| Type 1 | 5663 (2.6) | 6298 (0.5) |
| Type 2 | 4322 (2.0) | 91791 (7.0) |
| Hypertension, n (%) | 12127 (5.6) | 423290 (32.2) |
| Myocardial infarction, n (%) | 570 (0.3) | 62166 (4.7) |
| BMI (kg/m^2) | 29.0 (7.34) | 28.4 (5.81) |
| BMI category, n (%) |  |  |
| Underweight | 2587 (1.2) | 8019 (0.6) |
| Normal Weight | 27375 (12.7) | 154340 (11.7) |
| Overweight | 25155 (11.6) | 209382 (15.9) |
| Obese | 33623 (15.5) | 179222 (13.6) |
| Missing % | 59.0 | 58.1 |
| SBP (mmHg) | 127 (17.1) | 140 (20.4) |
| Missing % | 34.7 | 19.7 |
| DBP (mmHg) | 79.3 (11.9) | 82.2 (11.4) |
| Missing % | 34.7 | 19.7 |
| HDL (mmol/L), median (IQR) | 1.3 (1.10, 1.56) | 1.4 (1.15, 1.70) |
| Missing % | 10.5 | 11.6 |
| LDL (mmol/L), median (IQR) | 2.86 (2.3, 3.5) | 3.30 (2.6, 4.0) |
| Missing % | 17.9 | 20.8 |
| Total cholesterol (mmol/L), median (IQR) | 4.8 (4.2, 5.60) | 5.5 (4.7, 6.22) |
| Missing % | 2.3 | 1.9 |
| Triglycerides (mmol/L), median (IQR) | 1.11 (0.80, 1.70) | 1.35 (0.96, 1.96) |
| Triglycerides category, n (%) |  |  |
| ≤1.7 | 163325 (75.5) | 890458 (67.8) |
| (1.7,4.5] | 48194 (22.3) | 395574 (30.1) |
| (4.5,10] | 4183 (1.9) | 25418 (1.9) |
| (10,20] | 512 (0.2) | 2157 (0.2) |
| >20 | 148 (0.1) | 472 (0.04) |
| Missing % | 0 | 0 |
| Statin, n (%) | 5125 (2.4) | 236676 (18) |
| Fibrate, n (%) | 348 (0.2) | 15543 (1.2) |
| Statin + fibrate, n (%) | 106 (0.05) | 2688 (0.2) |
| Nicotinic acid, n (%) | 17 (0.008) | 528 (0.04) |
| Omega-3 fatty acids, n (%) | 171 (0.1) | 2569 (0.2) |
| Other lipid lowering, n (%) | 395 (0.2) | 11567 (0.9) |
| Follow-up time (years), median (IQR) | 5.32 (2.53, 9.01) | 6.90 (3.55, 10.67) |

**Supplementary Table 2: Hazard ratios for all variables in the multivariable models**

| **Variable** | **All-cause mortality** | **Acute pancreatitis** | **Chronic pancreatitis** | **New-onset diabetes** | **Myocardial infarction** |
| --- | --- | --- | --- | --- | --- |
| Age (per 10 years) | 2.84 (2.82, 2.85) | 1.29 (1.27, 1.32) | 1.07 (1.04, 1.11) | 1.37 (1.36, 1.38) | 1.90 (1.89, 1.91) |
| Men | 1.29 (1.28, 1.30) | 1.05 (0.99, 1.10) | 1.58 (1.45, 1.73) | 1.19 (1.17, 1.20) | 1.82 (1.78, 1.85) |
| Smoking status (vs. non-smoker) |  |  |  |  |  |
| Ex-smoker | 1.32 (1.31, 1.34) | 1.19 (1.13, 1.25) | 1.74 (1.56, 1.93) | 1.10 (1.09, 1.12) | 1.36 (1.34, 1.38) |
| Smoker | 2.14 (2.11, 2.18) | 1.68 (1.57, 1.79) | 3.64 (3.29, 4.02) | 1.25 (1.23, 1.27) | 1.88 (1.84, 1.93) |
| Alcohol consumption (vs. occasional drinker) |  |  |  |  |  |
| Non-drinker | 1.14 (1.12, 1.17) | 1.18 (1.08, 1.29) | 1.29 (1.08, 1.55) | 1.13 (1.10, 1.16) | 1.11 (1.07, 1.15) |
| Ex-drinker | 1.43 (1.37, 1.50) | 2.22 (1.78, 2.78) | 3.30 (2.64, 4.13) | 1.03 (0.97, 1.10) | 1.15 (1.08, 1.23) |
| Current drinker | 0.90 (0.89, 0.92) | 0.94 (0.87, 1.01) | 0.81 (0.69, 0.96) | 0.88 (0.86, 0.90) | 0.86 (0.84, 0.89) |
| Excess drinker | 1.17 (1.14, 1.20) | 1.24 (1.13, 1.36) | 2.02 (1.69, 2.42) | 0.90 (0.87. 0.93) | 0.85 (0.81, 0.88) |
| Diabetes type (vs. no diabetes) |  |  |  |  |  |
| Type 2 diabetes | 1.31 (1.29, 1.33) | 1.24 (1.14, 1.33) | 2.27 (2.03, 2.55) | - | 1.12 (1.10, 1.15) |
| Type 1 diabetes | 2.56 (2.42, 2.70) | 1.80 (1.43, 2.25) | 4.93 (4.02, 6.04) | - | 2.00 (1.86, 2.15) |
| Unspecified type | 3.02 (2.86, 3.20) | 3.00 (2.28, 3.94) | 8.41 (6.32, 11.19) | - | 2.41 (2.21, 2.62) |
| Hypertension | 1.17 (1.16, 1.18) | 1.17 (1.11, 1.23) | 1.32 (1.21, 1.45) | 1.27 (1.26, 1.29) | 1.19 (1.17, 1.20) |
| Triglyceride group vs. [0.01, 1.7] (mmol/L) |  |  |  |  |  |
| (1.7,4.5] | 1.08 (1.07, 1.10) | 1.31 (1.25, 1.39) | 1.41 (1.29, 1.54) | 1.74 (1.72, 1.76) | 1.07 (1.05, 1.09) |
| (4.5,10] | 1.43 (1.38, 1.48) | 2.05 (1.81, 2.32) | 3.22 (2.69, 3.86) | 2.75 (2.67, 2.83) | 1.17 (1.12, 1.23) |
| (10,20] | 2.02 (1.79, 2.29) | 4.31 (3.24, 5.72) | 6.47 (4.33, 9.67) | 3.33 (3.08, 3.60) | 1.03 (0.88, 1.19) |
| (20,50] | 3.62 (2.82, 4.65) | 13.55 (9.15, 20.06) | 25.19 (14.91, 42.55) | 5.28 (4.51, 6.18) | 1.02 (0.75, 1.40) |
| BMI (per kg/m^2^) | 0.991 (0.990, 0.992) | 1.024 (1.018, 1.030) | 0.927 (0.913, 0.942) | 1.062 (1.061, 1.063) | 0.996 (0.994, 0.998) |
| HDL (per mmol/L) | 0.955 (0.941, 0.970) | 1.033 (0.971, 1.100) | 1.113 (1.067, 1.160) | 0.503 (0.491, 0.515) | 0.610 (0.594, 0.626) |
| Total cholesterol (per mmol/L) | 0.893 (0.888, 0.897) | 0.926 (0.905, 0.946) | 0.827 (0.797, 0.858) | 0.979 (0.974, 0.984) | 1.025 (1.018, 1.032) |
| Prescribed medication |  |  |  |  |  |
| Statins | 1.06 (1.05, 1.07) | 0.96 (0.90, 1.02) | 0.74 (0.66, 0.82) | 1.09 (1.07, 1.11) | 2.76 (2.72, 2.80) |
| Fibrates | 1.17 (1.13, 1.21) | 1.38 (1.19, 1.60) | 1.78 (1.42, 2.23) | 1.23 (1.18, 1.27) | 1.20 (1.15, 1.24) |
| Nicotinic acid | 1.11 (0.94, 1.32) | 1.84 (1.01, 3.35) | 2.93 (1.42, 6.05) | 1.11 (0.91, 1.36) | 0.99 (0.81, 1.22) |
| Omega-3 fatty acids | 0.99 (0.89, 1.09) | 1.42 (0.96, 2.10) | 1.78 (1.08, 2.92) | 0.99 (0.89, 1.11) | 2.51 (2.32, 2.71) |
| Other lipid lowering drugs | 1.12 (1.07, 1.17) | 1.43 (1.18, 1.73) | 2.20 (1.69, 2.87) | 0.91 (0.86, 0.96) | 1.26 (1.19, 1.32) |

**Supplementary Figure 1: Kaplan Meier Curves for association of triglycerides and clinical outcomes stratified by age >40 and <=40 years**

| **Age Stratification** |  |
| --- | --- |
| **(A)Acute Pancreatitis**  **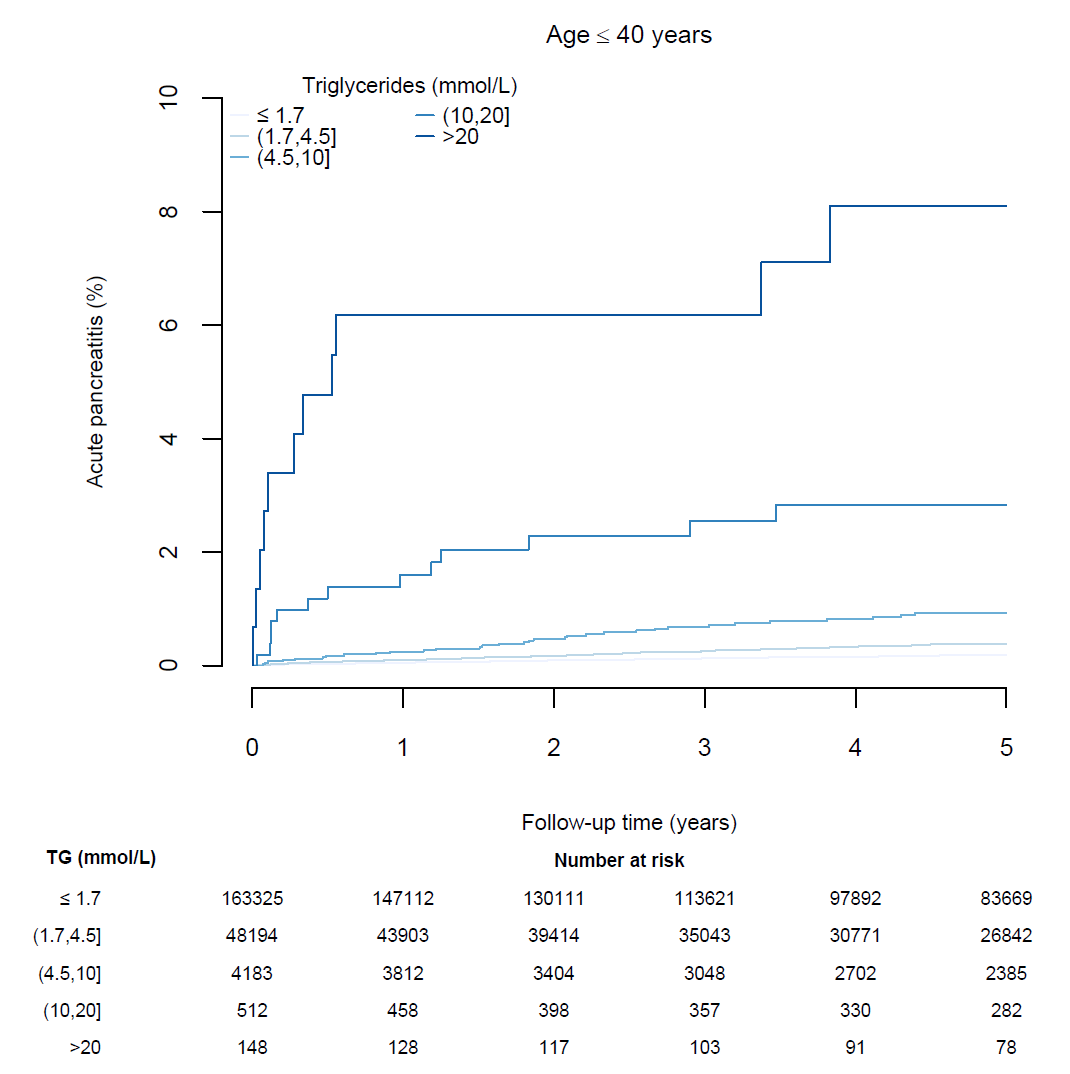** | **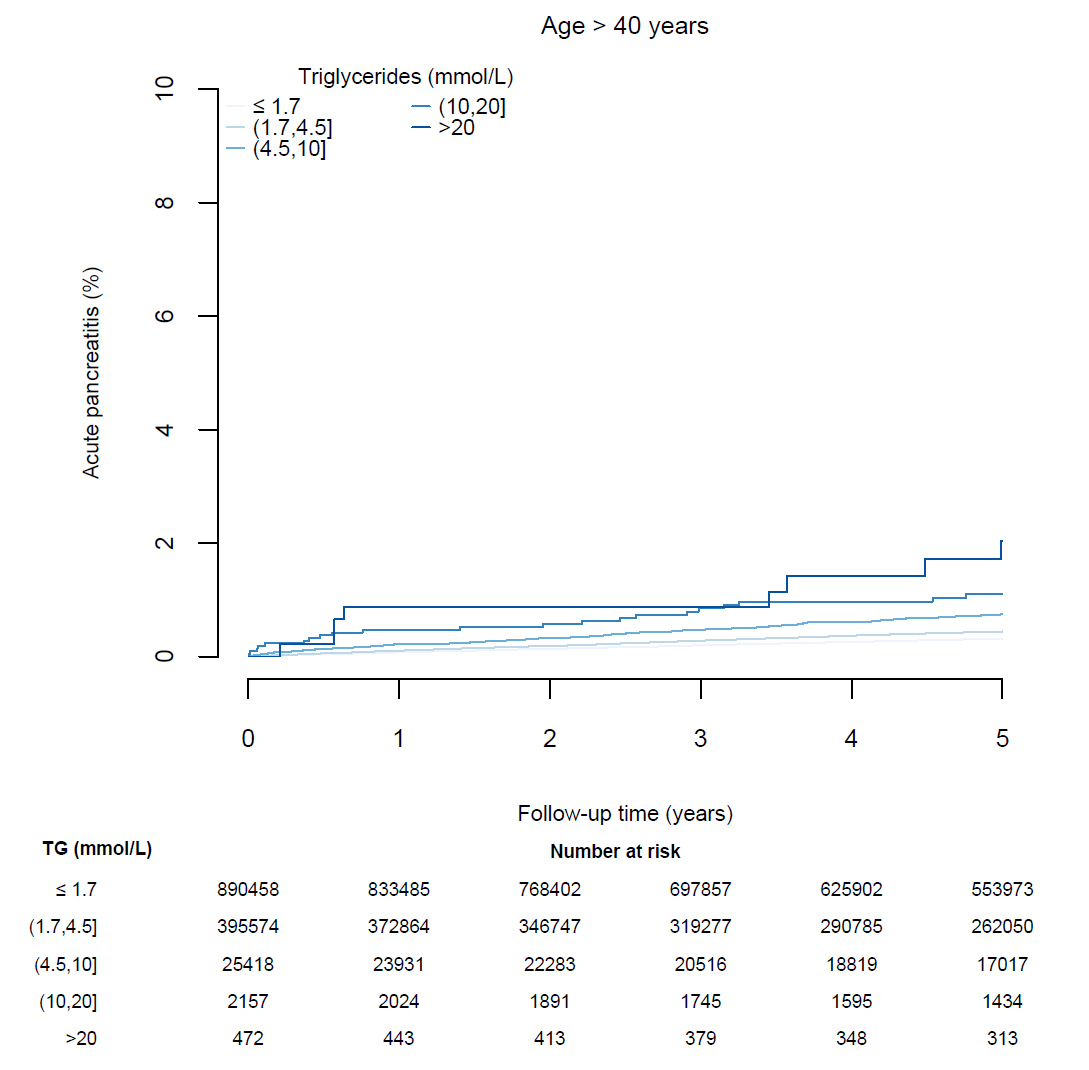** |
| **(B)Chronic Pancreatitis**  **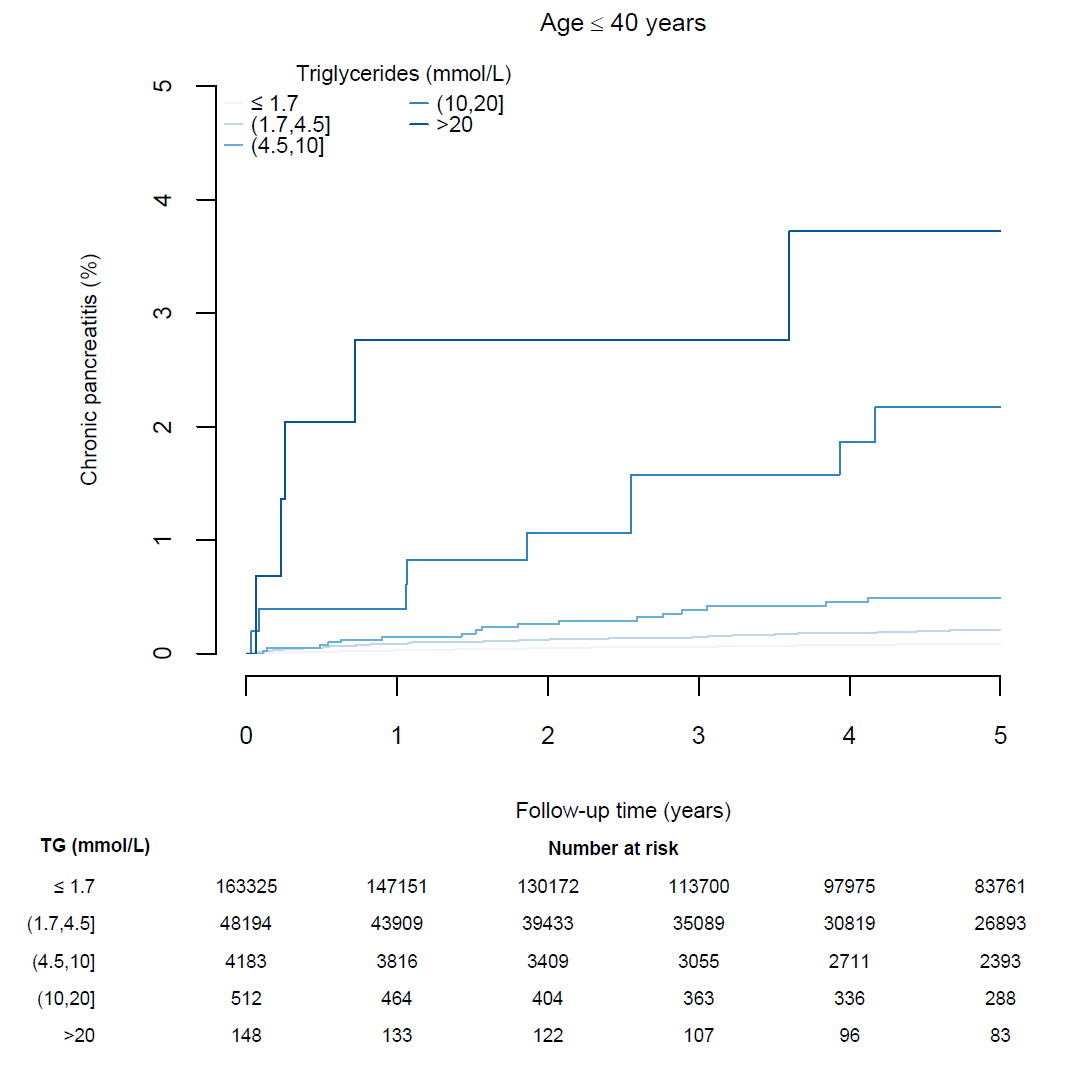** | **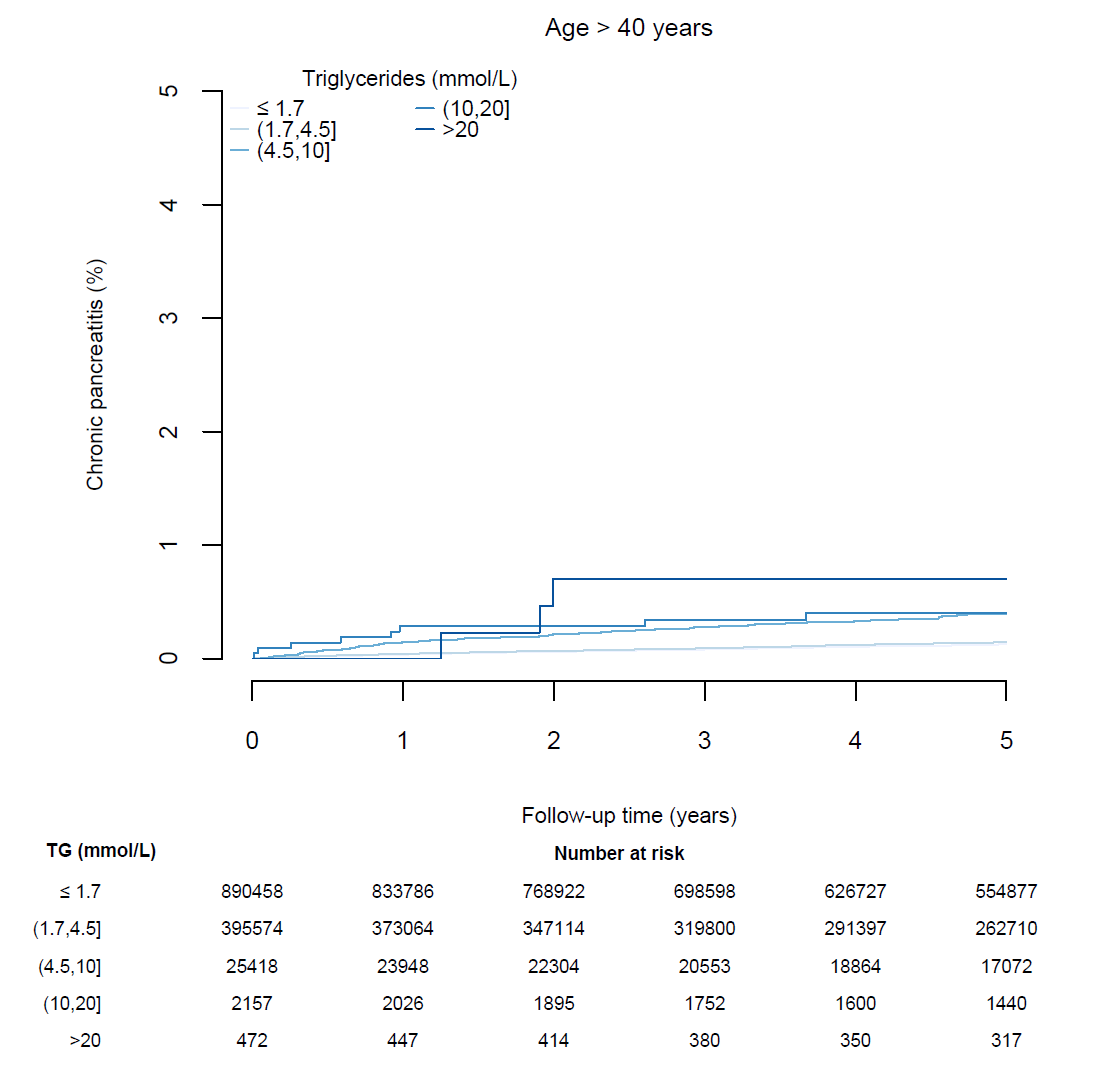** |
| **(C)Diabetes**  **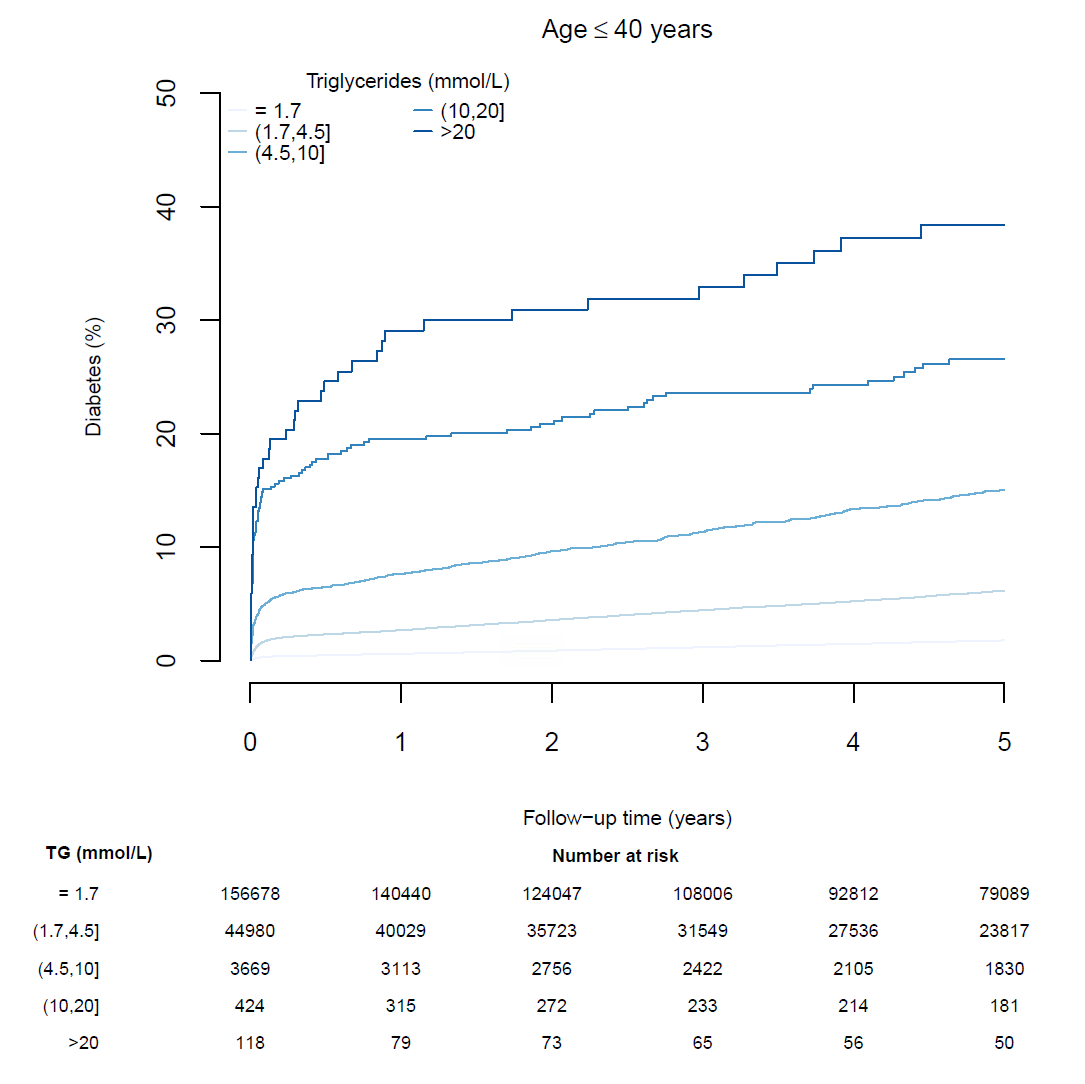** | **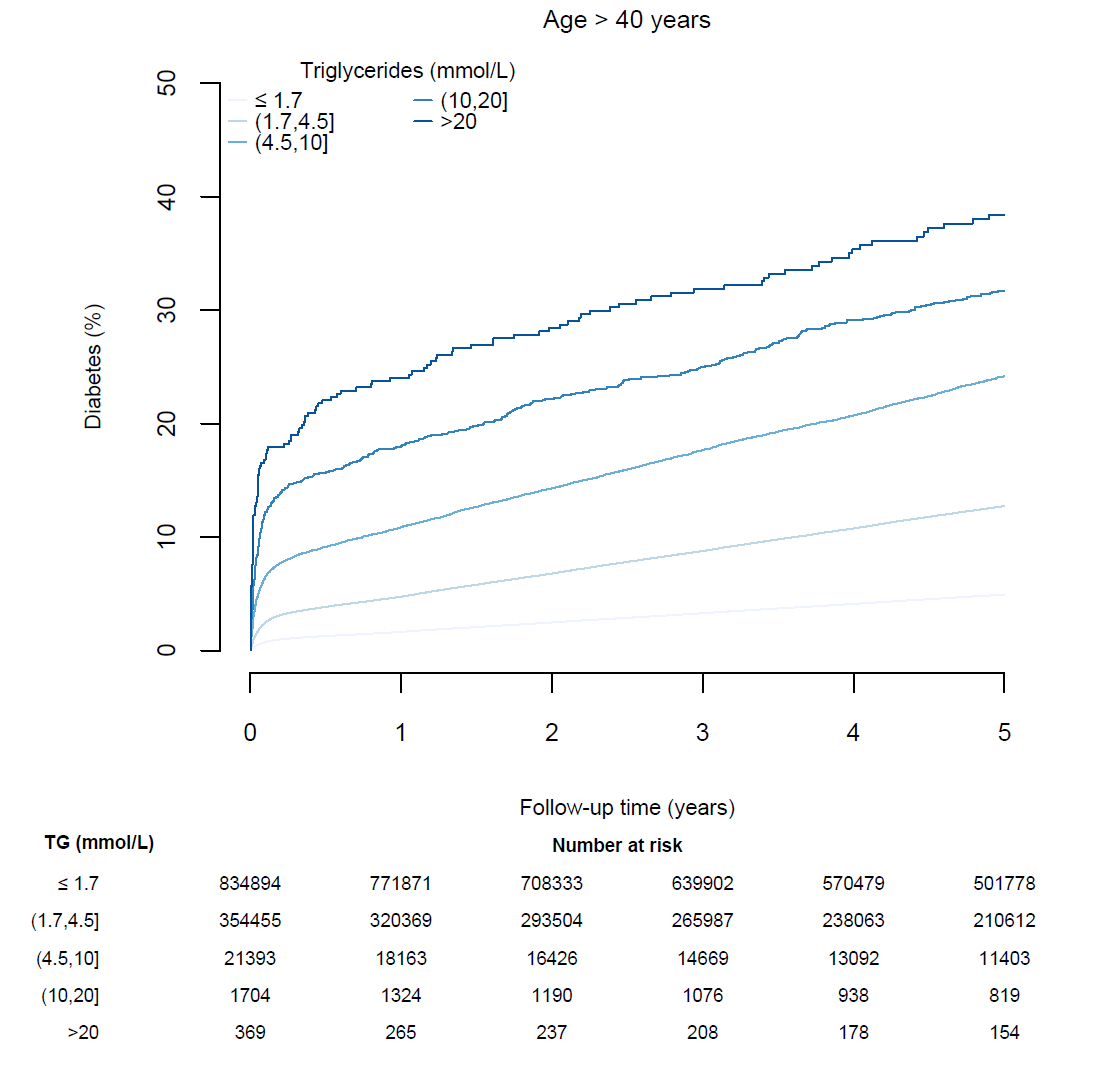** |
| **(D)Myocardial Infarction**  **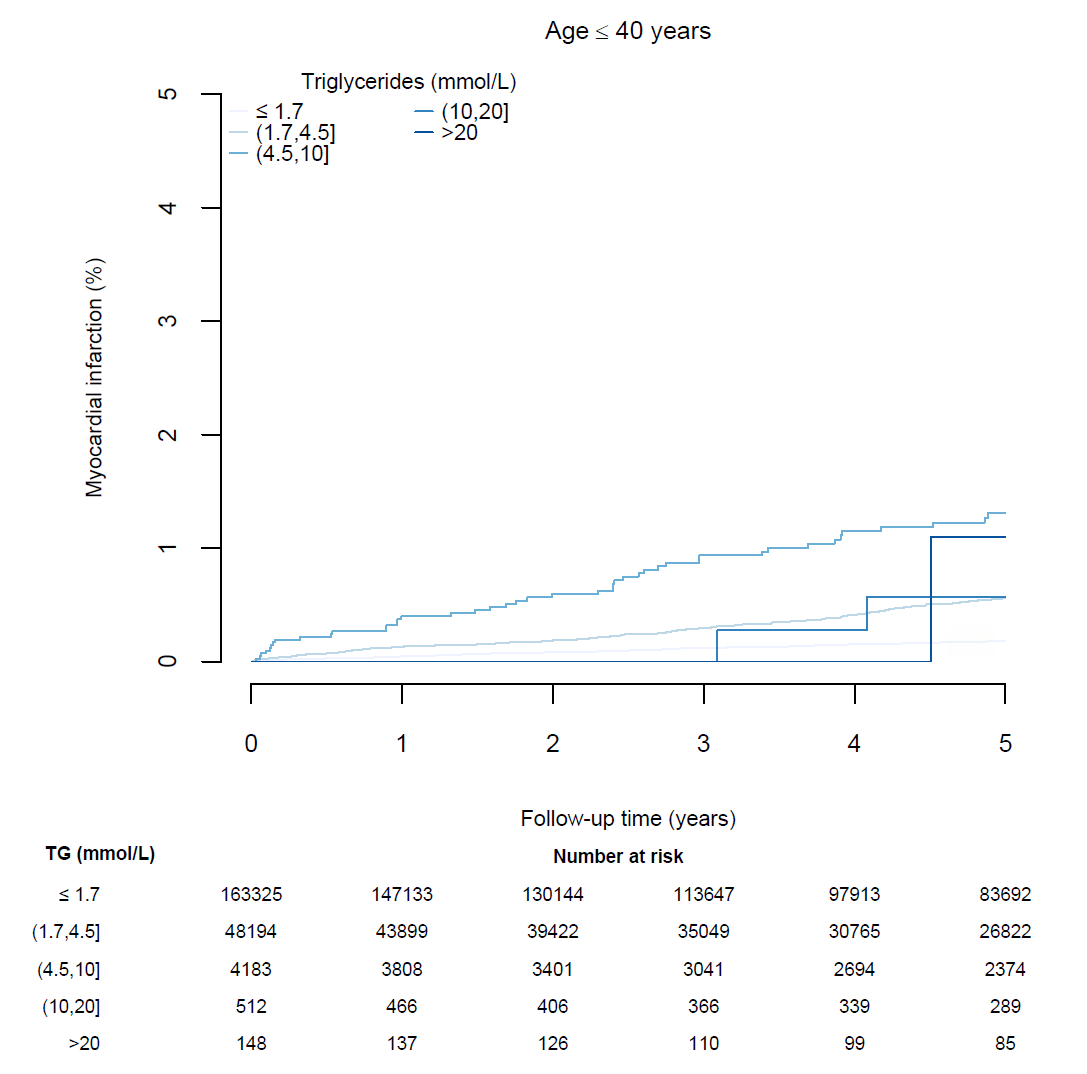** | **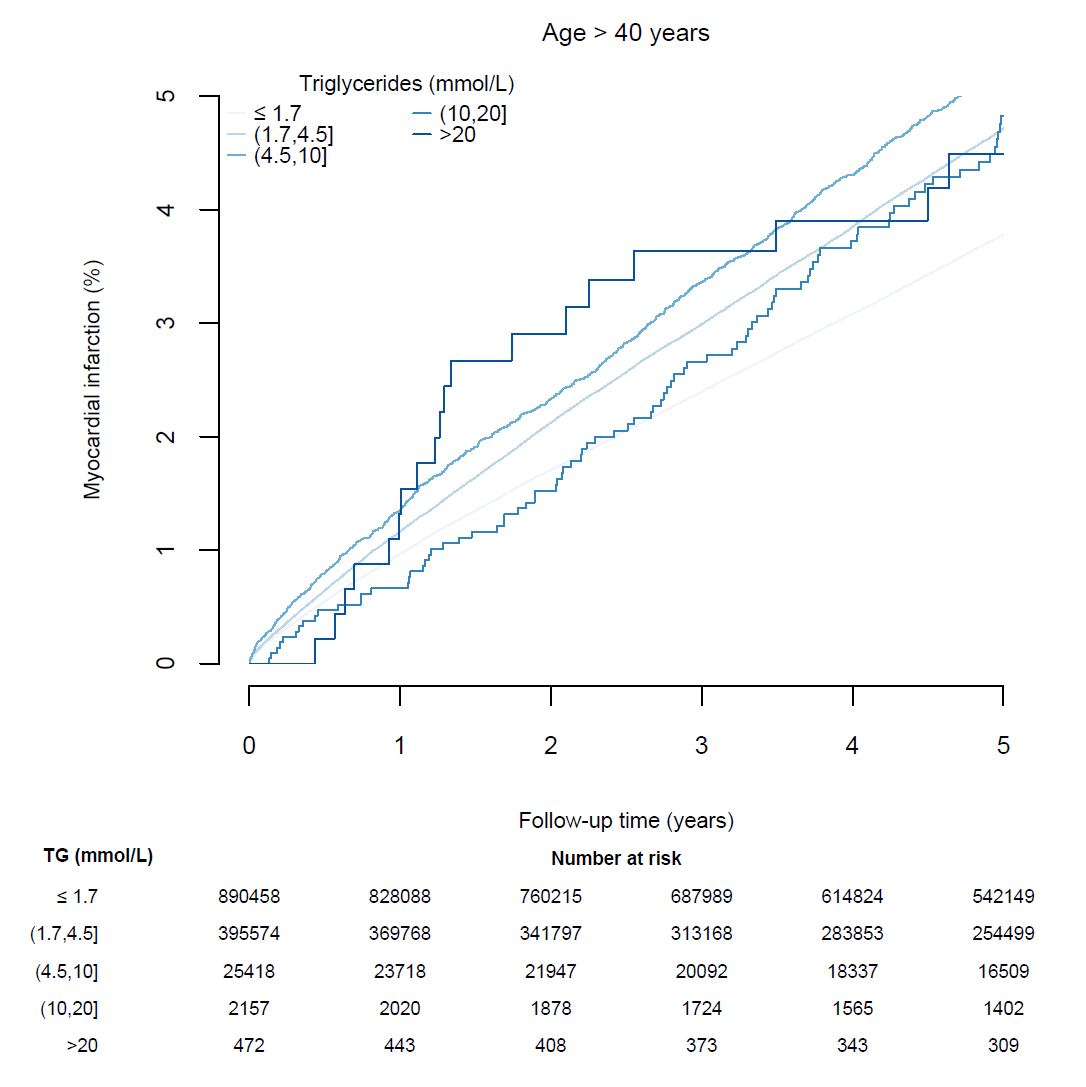** |
| **(E)All-Cause Mortality**  **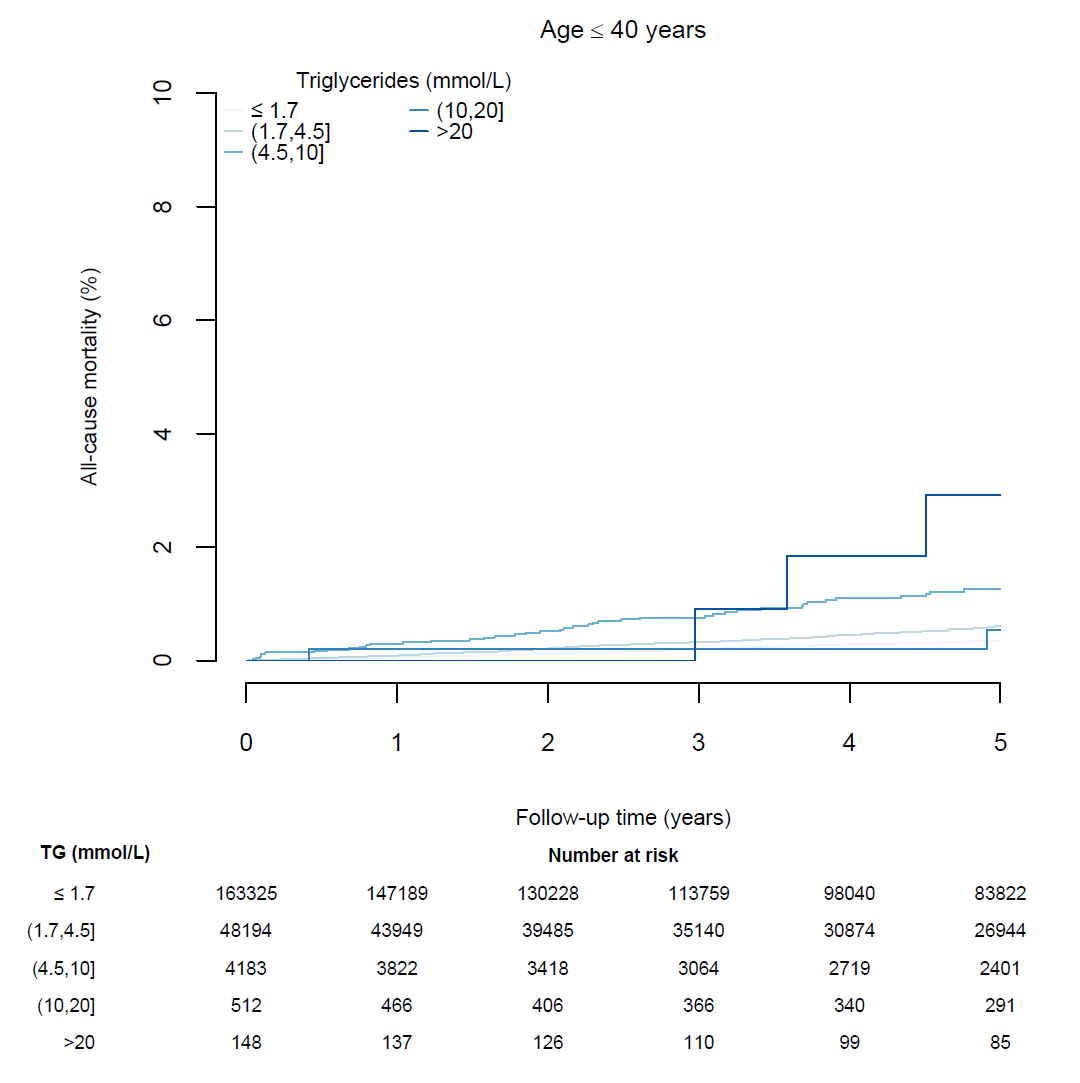** | **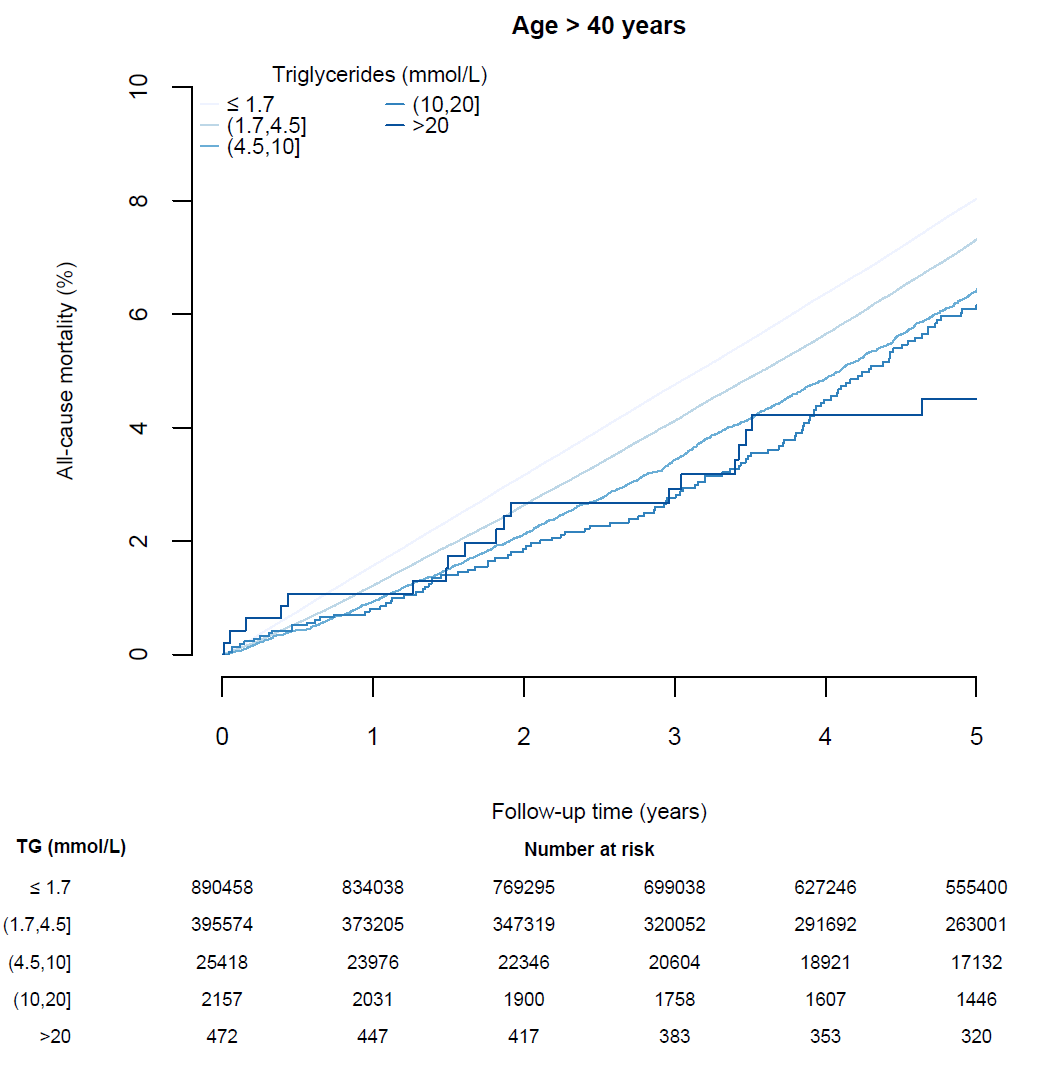** |

**Supplementary table 3: Kaplan-Meier event estimates (%) at 1 and 5 years for each clinical outcome stratified by baseline TG and age group**

|  |  |  | Baseline TG (mmol/L) | | | | |
| --- | --- | --- | --- | --- | --- | --- | --- |
| Endpoint | Age group | Time (years) | ≤1.7 | (1.7,4.5] | (4.5,10] | (10,20] | >20 |
| Acute pancreatitis | ≤40 | 1 | 0.05 | 0.10 | 0.25 | 1.60 | 6.18 |
|  |  | 5 | 0.19 | 0.39 | 0.93 | 2.83 | 8.09 |
|  | >40 | 1 | 0.07 | 0.10 | 0.21 | 0.47 | 0.87 |
|  |  | 5 | 0.32 | 0.44 | 0.75 | 1.10 | 2.03 |
| Chronic pancreatitis | ≤40 | 1 | 0.03 | 0.09 | 0.15 | 0.39 | 2.77 |
|  |  | 5 | 0.09 | 0.21 | 0.49 | 2.17 | 3.72 |
|  | >40 | 1 | 0.04 | 0.04 | 0.15 | 0.29 | 0.00 |
|  |  | 5 | 0.13 | 0.14 | 0.39 | 0.40 | 0.70 |
| New onset diabetes | ≤40 | 1 | 0.60 | 2.69 | 7.65 | 19.48 | 29.05 |
|  |  | 5 | 1.78 | 6.14 | 14.99 | 26.53 | 38.43 |
|  | >40 | 1 | 1.65 | 4.75 | 10.87 | 17.97 | 24.04 |
|  |  | 5 | 4.94 | 12.73 | 24.16 | 31.65 | 38.41 |
| Myocardial infarction | ≤40 | 1 | 0.05 | 0.13 | 0.40 | 0.00 | 0.00 |
|  |  | 5 | 0.18 | 0.56 | 1.31 | 0.57 | 1.10 |
|  | >40 | 1 | 0.96 | 1.16 | 1.35 | 0.66 | 1.32 |
|  |  | 5 | 3.78 | 4.71 | 5.28 | 4.82 | 4.49 |
| All-cause mortality | ≤40 | 1 | 0.06 | 0.09 | 0.30 | 0.20 | 0.00 |
|  |  | 5 | 0.35 | 0.61 | 1.25 | 0.54 | 2.92 |
|  | >40 | 1 | 1.57 | 1.22 | 0.93 | 0.80 | 1.07 |
|  |  | 5 | 8.03 | 7.32 | 6.42 | 6.16 | 4.51 |

**Supplementary figure 2: Hazard ratios for triglyceride values and clinical outcomes, adjusted for age and sex and stratified by Age ≤ 40 or > 40 years**

**
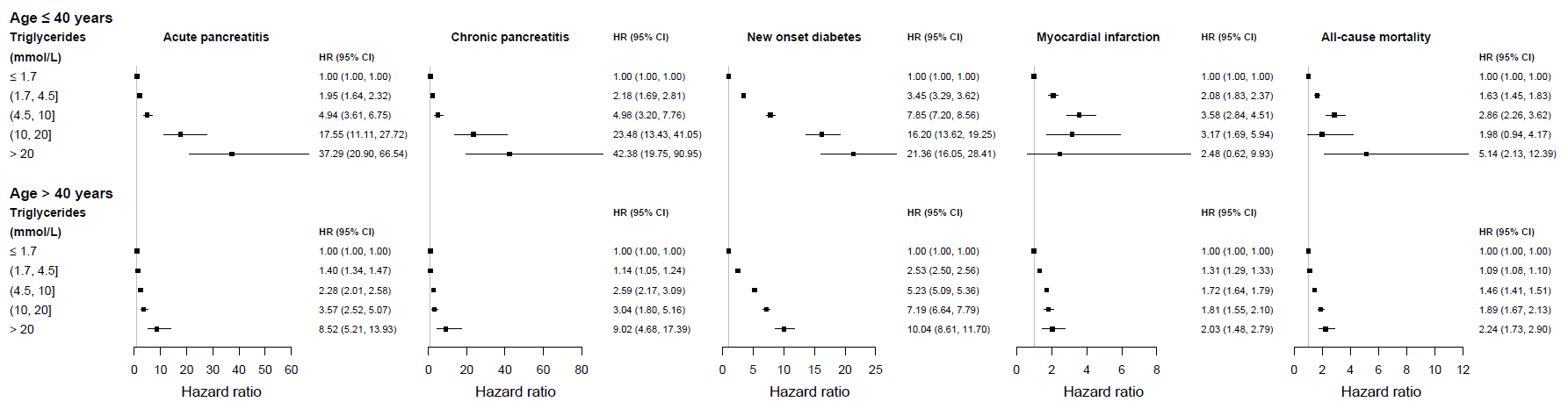
**

**Supplementary Figure 3: Kaplan Meier curves for association of triglycerides and clinical outcomes stratified by prior pancreatitis**

| **Prior Pancreatitis** | **No Prior Pancreatitis** |
| --- | --- |
| **(A)Acute Pancreatitis**  **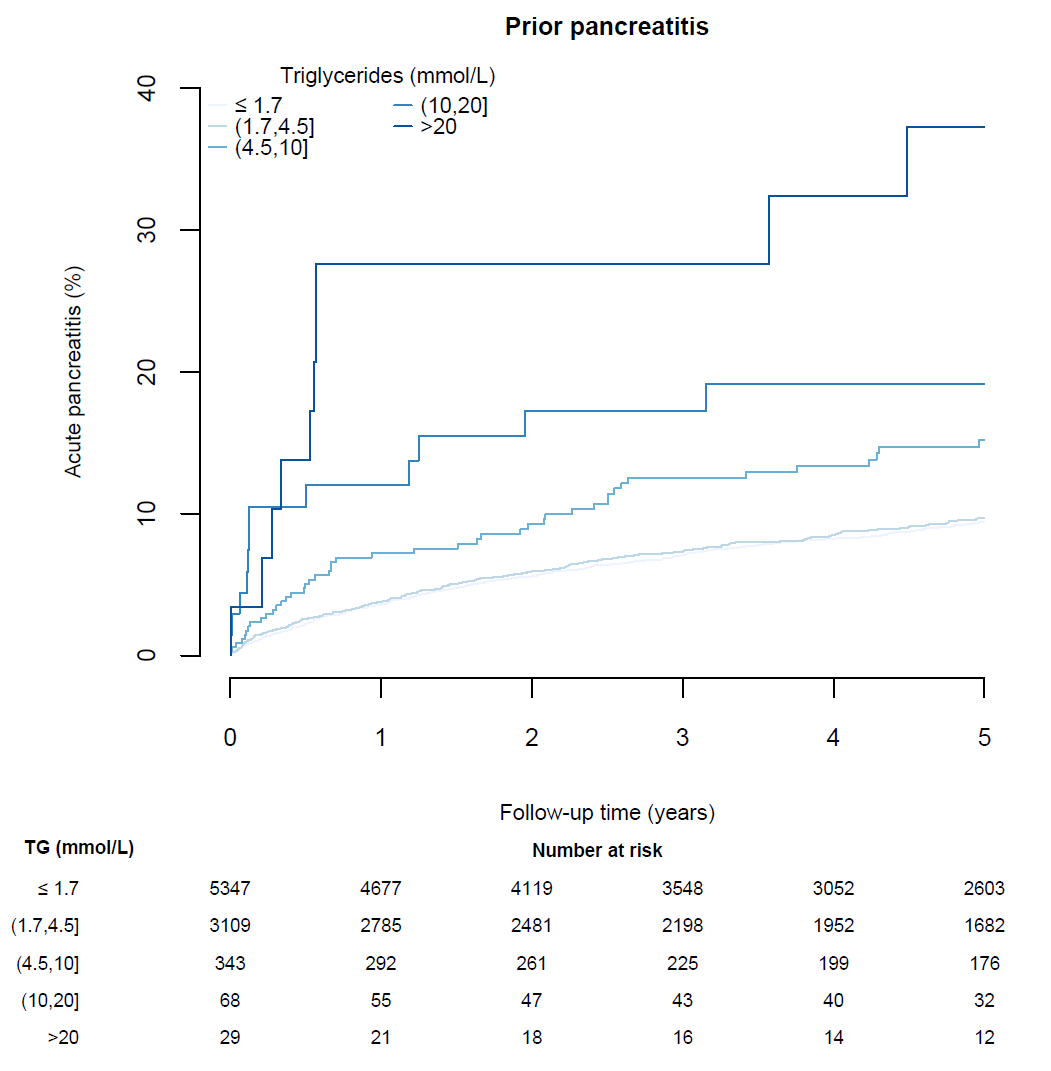** | **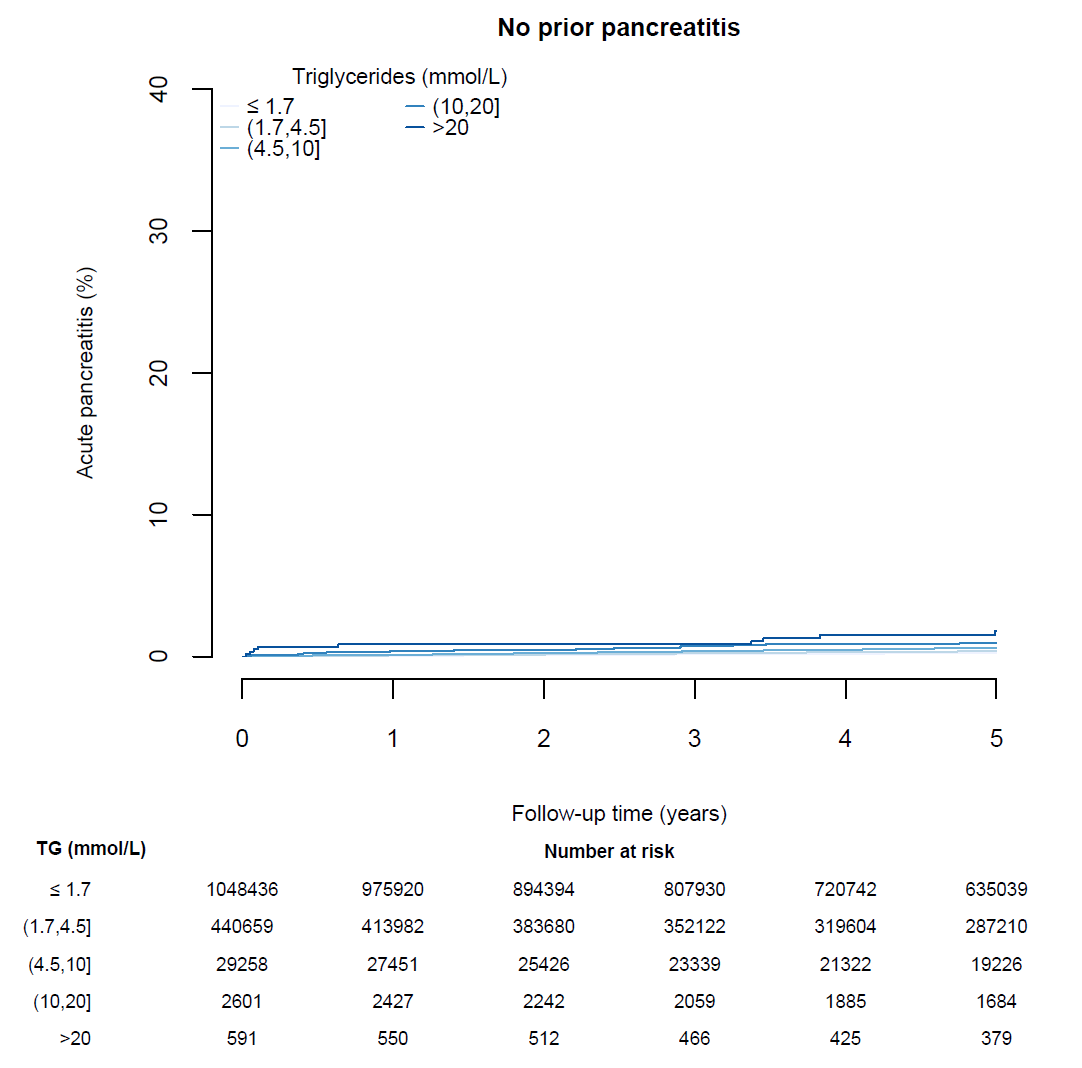** |
| **(B)Chronic Pancreatitis**  **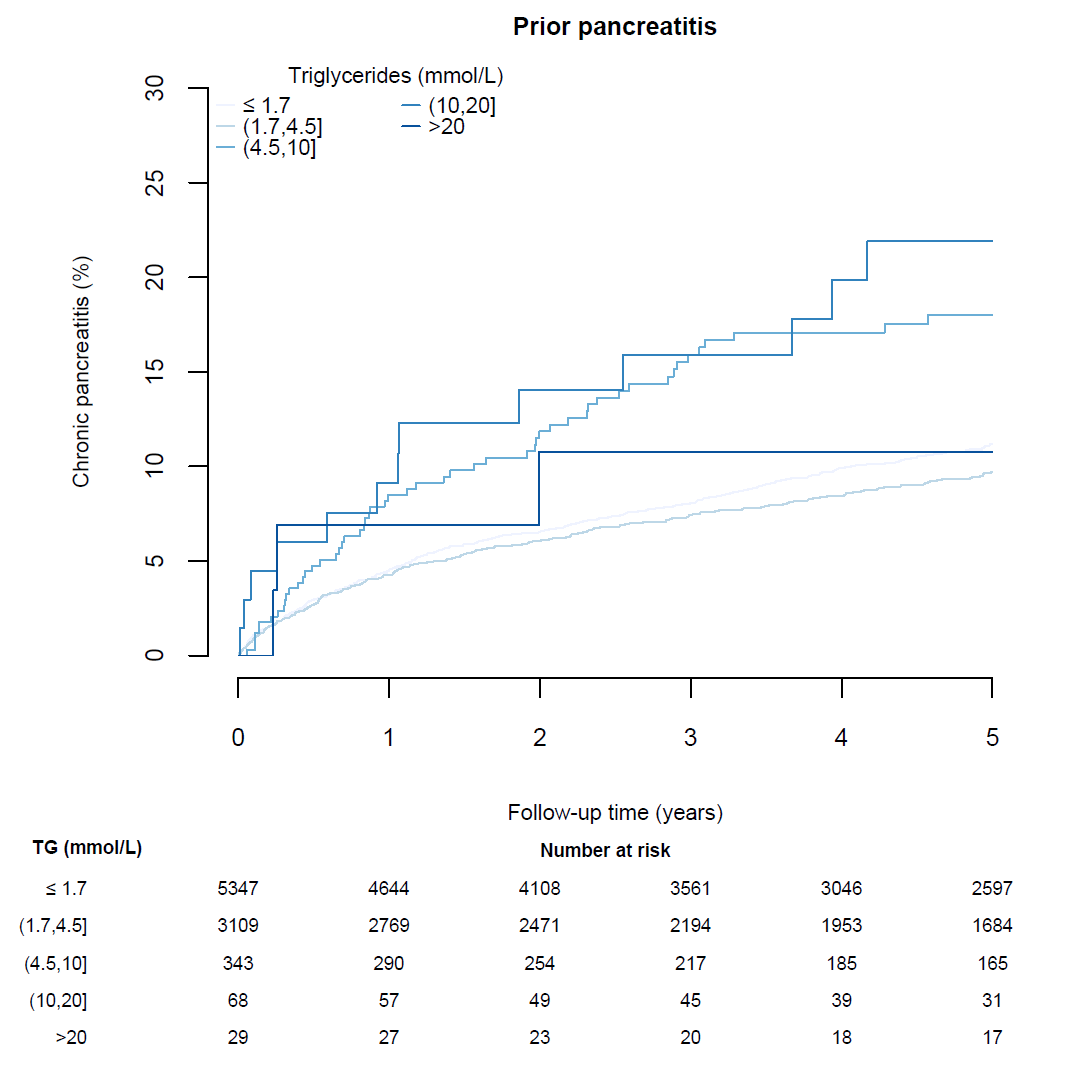** | **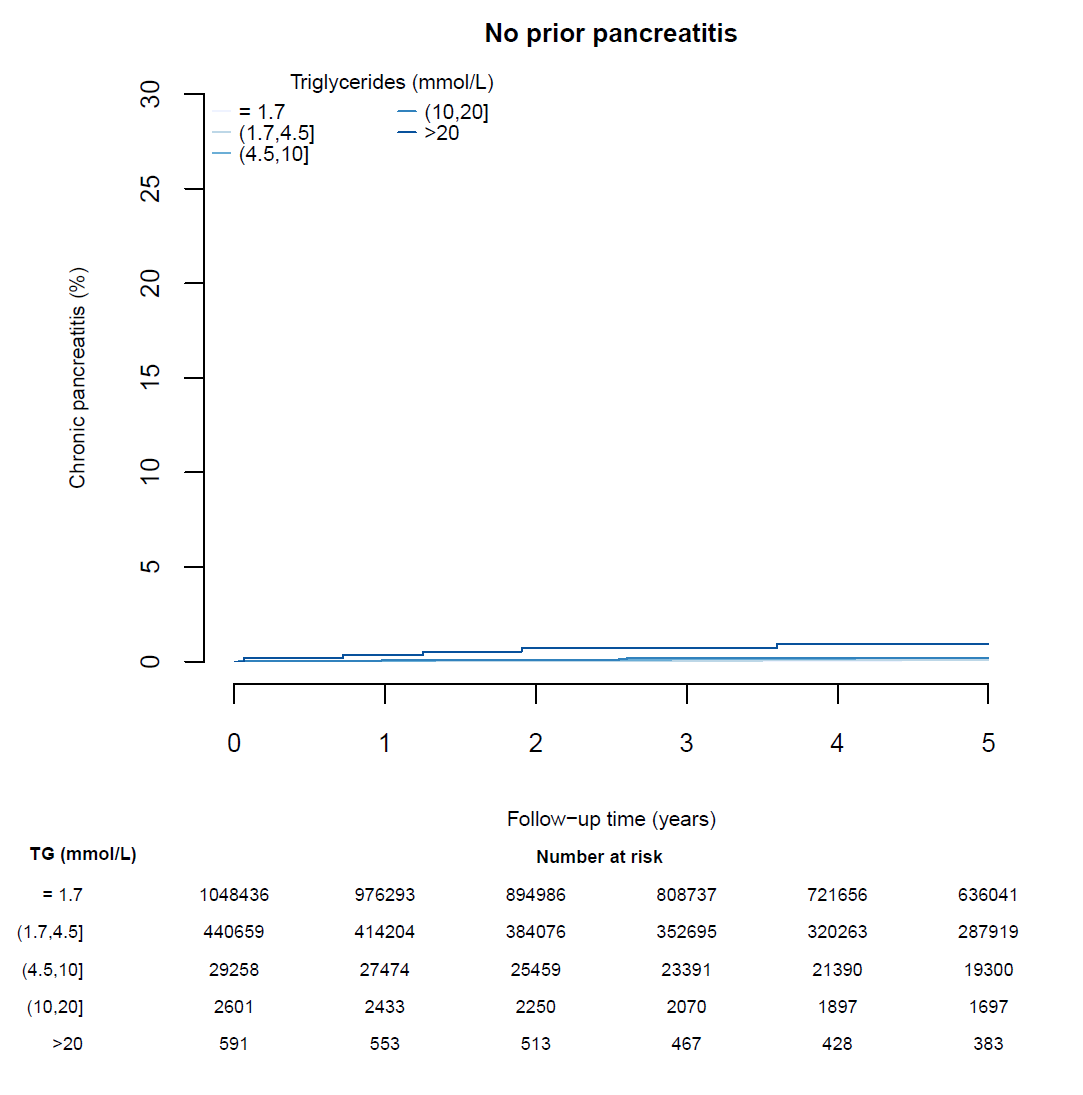** |
| **(C)Diabetes**  **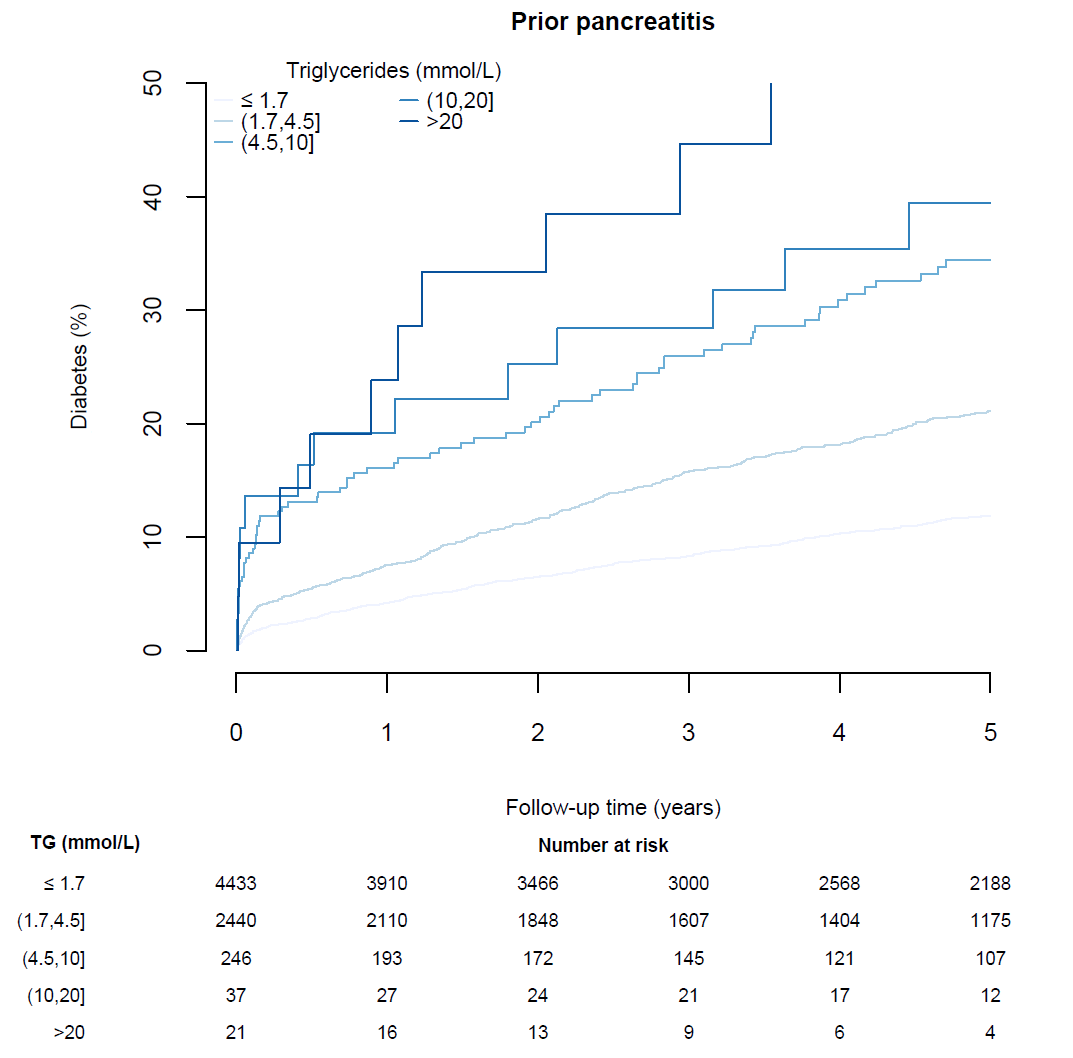** | **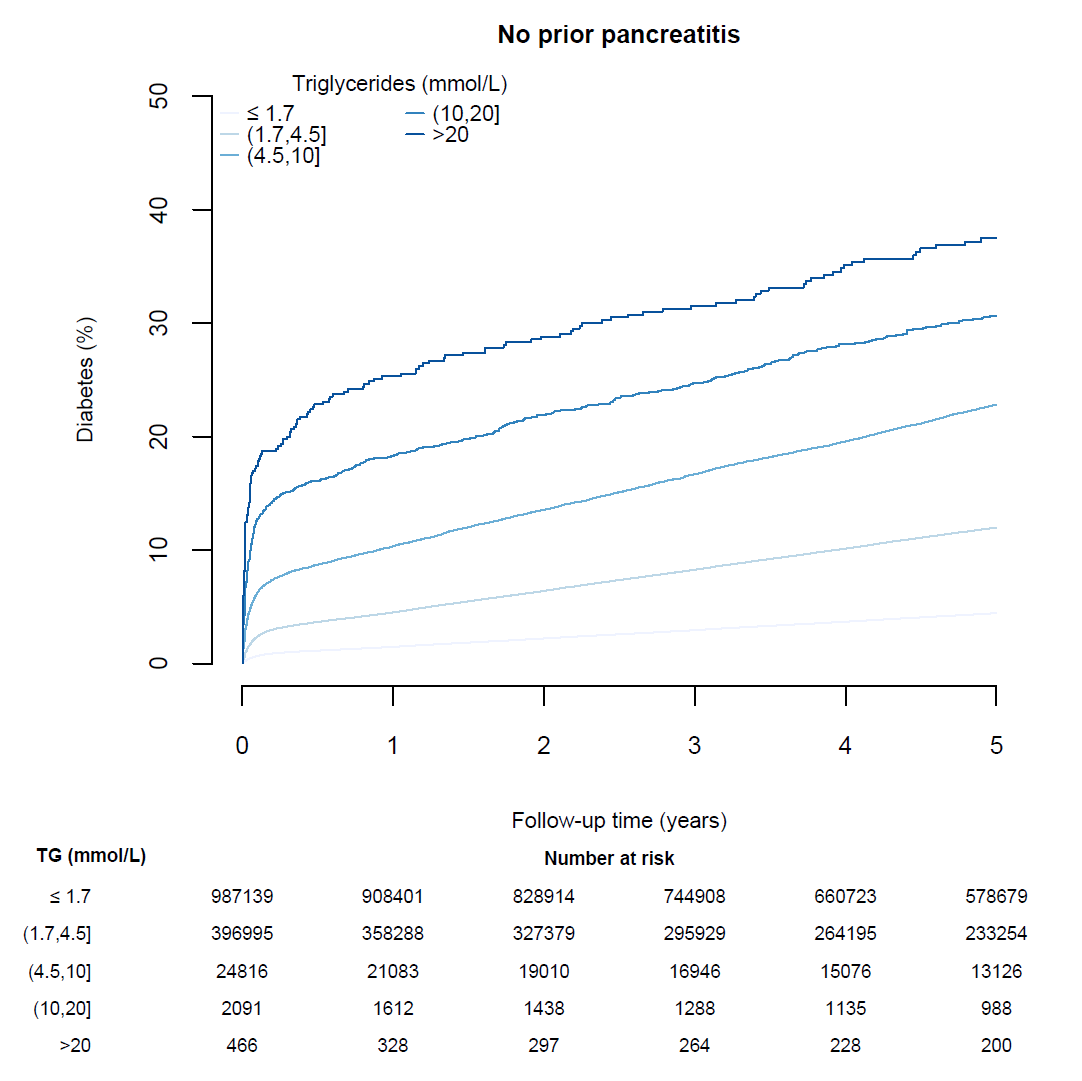** |
| **(D)Myocardial Infarction**  **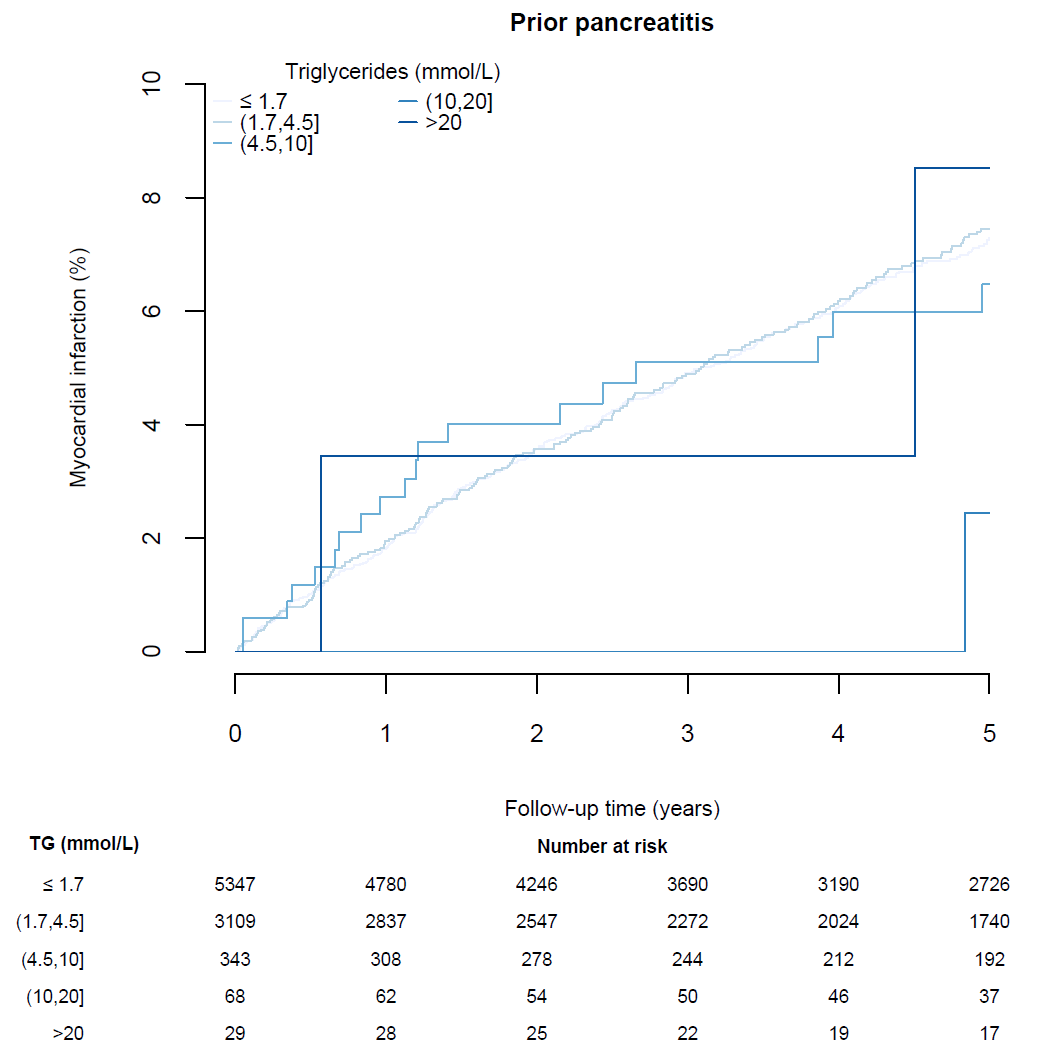** | **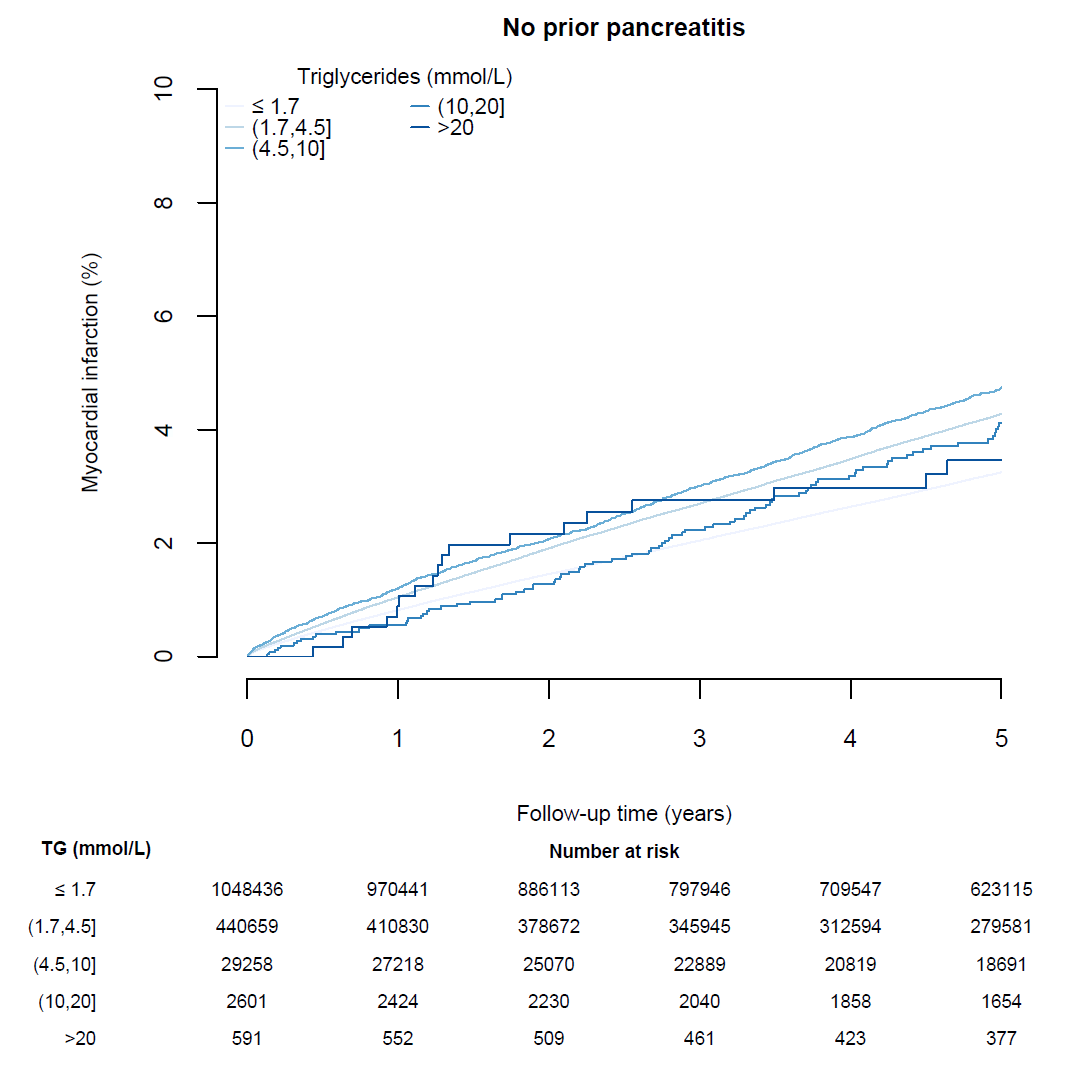** |
| **(E)All-Cause Mortality**  **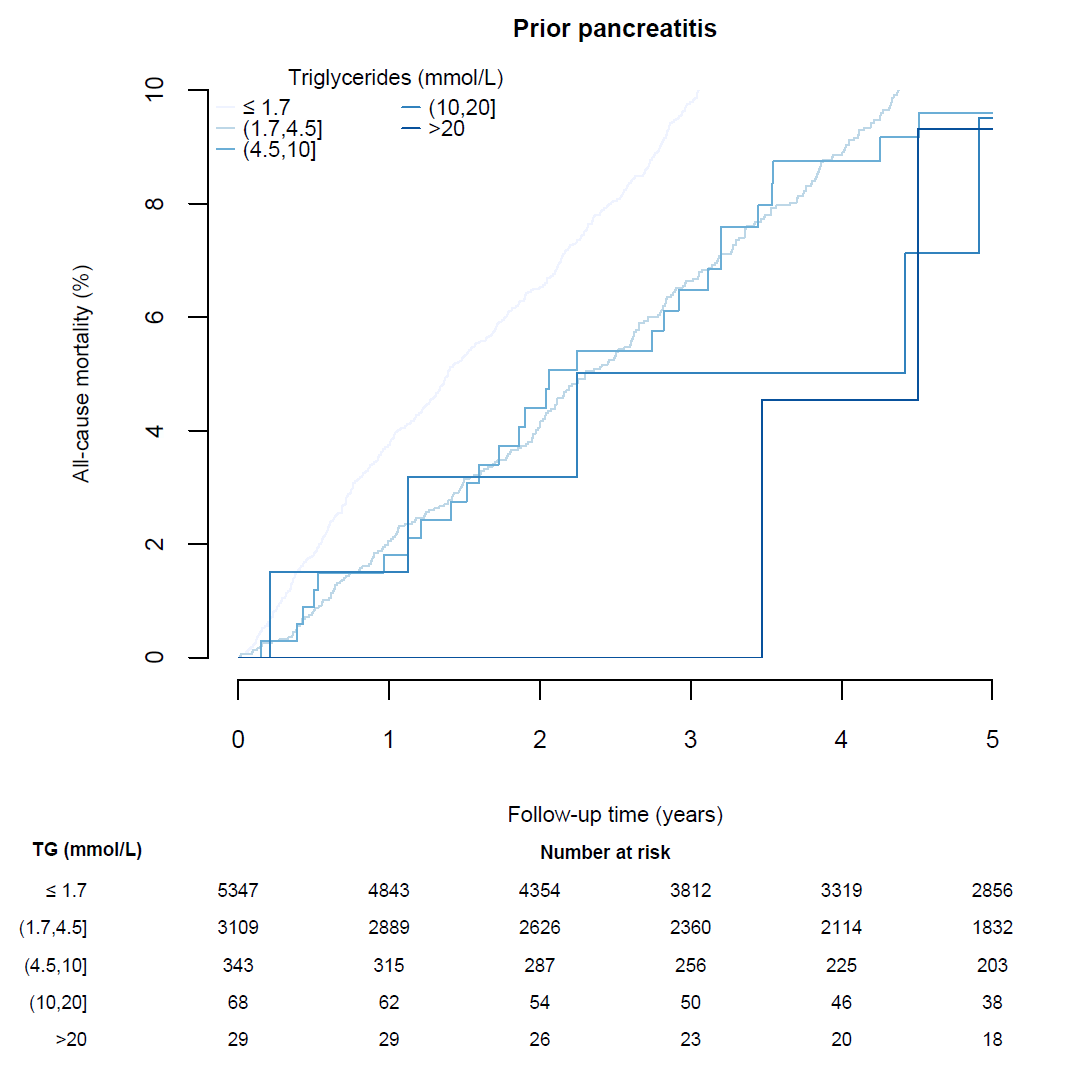** | **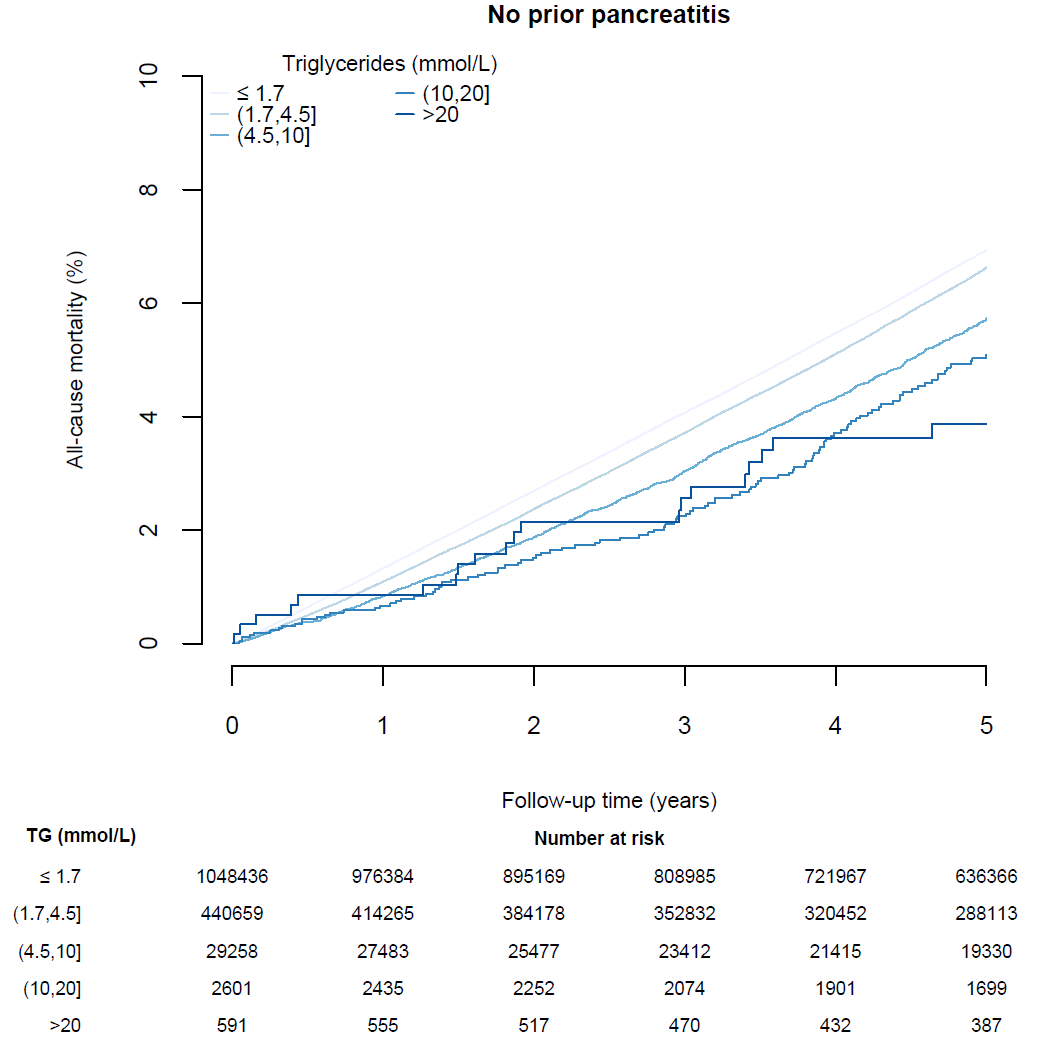** |

**Supplementary Table 4: Kaplan-Meier point estimates at 1 and 5 years for each clinical outcome stratified by baseline TG and prior pancreatitis**

|  |  |  | Baseline TG (mmol/L) | | | | |
| --- | --- | --- | --- | --- | --- | --- | --- |
| Clinical outcomes | Prior pancreatitis | Time (years) | ≤1.7 | (1.7,4.5] | (4.5,10] | (10,20] | >20 |
| Acute pancreatitis | No | 1 | 0.05 | 0.08 | 0.14 | 0.39 | 0.86 |
|  |  | 5 | 0.26 | 0.37 | 0.61 | 0.97 | 1.78 |
|  | Yes | 1 | 3.61 | 3.78 | 7.22 | 12.02 | 27.59 |
|  |  | 5 | 9.41 | 9.73 | 15.20 | 19.16 | 37.24 |
| Chronic pancreatitis | No | 1 | 0.01 | 0.02 | 0.05 | 0.08 | 0.35 |
|  |  | 5 | 0.07 | 0.09 | 0.21 | 0.17 | 0.94 |
|  | Yes | 1 | 4.53 | 4.28 | 8.51 | 9.11 | 6.90 |
|  |  | 5 | 11.18 | 9.71 | 18.03 | 21.90 | 10.78 |
| New onset diabetes | No | 1 | 1.47 | 4.51 | 10.35 | 18.26 | 25.31 |
|  |  | 5 | 4.44 | 11.98 | 22.80 | 30.60 | 37.50 |
|  | Yes | 1 | 4.16 | 7.51 | 16.08 | 19.17 | 23.81 |
|  |  | 5 | 11.90 | 21.11 | 34.40 | 39.43 | 59.62 |
| Myocardial infarction | No | 1 | 0.82 | 1.04 | 1.20 | 0.55 | 0.89 |
|  |  | 5 | 3.25 | 4.28 | 4.74 | 4.12 | 3.46 |
|  | Yes | 1 | 1.83 | 1.96 | 2.73 | 0.00 | 3.45 |
|  |  | 5 | 7.29 | 7.46 | 6.47 | 2.44 | 8.53 |
| All-cause mortality | No | 1 | 1.33 | 1.09 | 0.83 | 0.67 | 0.86 |
|  |  | 5 | 6.93 | 6.63 | 5.72 | 5.09 | 3.86 |
|  | Yes | 1 | 3.75 | 2.05 | 1.80 | 1.52 | 0.00 |
|  |  | 5 | 15.48 | 12.41 | 9.60 | 9.50 | 9.32 |

**Supplementary Figure 4: Hazard ratios for triglyceride values and clinical outcomes, adjusted for age and sex and stratified by prior pancreatitis**

**
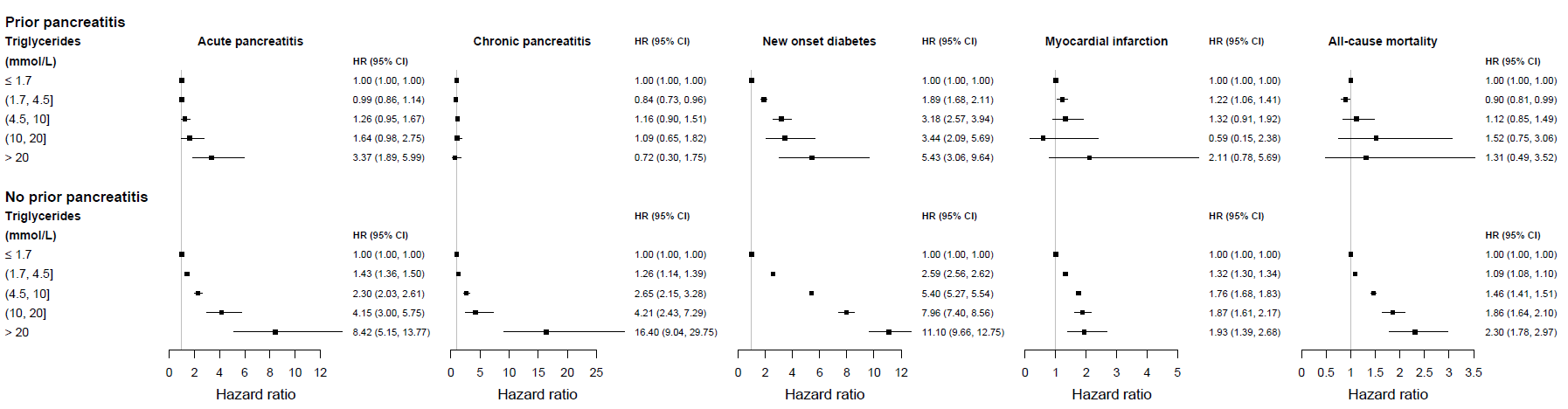
**

**Supplementary Figure 5: Multivariable adjusted association between triglycerides and each endpoint. Triglyceride level was fitted in the model using restricted cubic splines (3 knot points)**

**
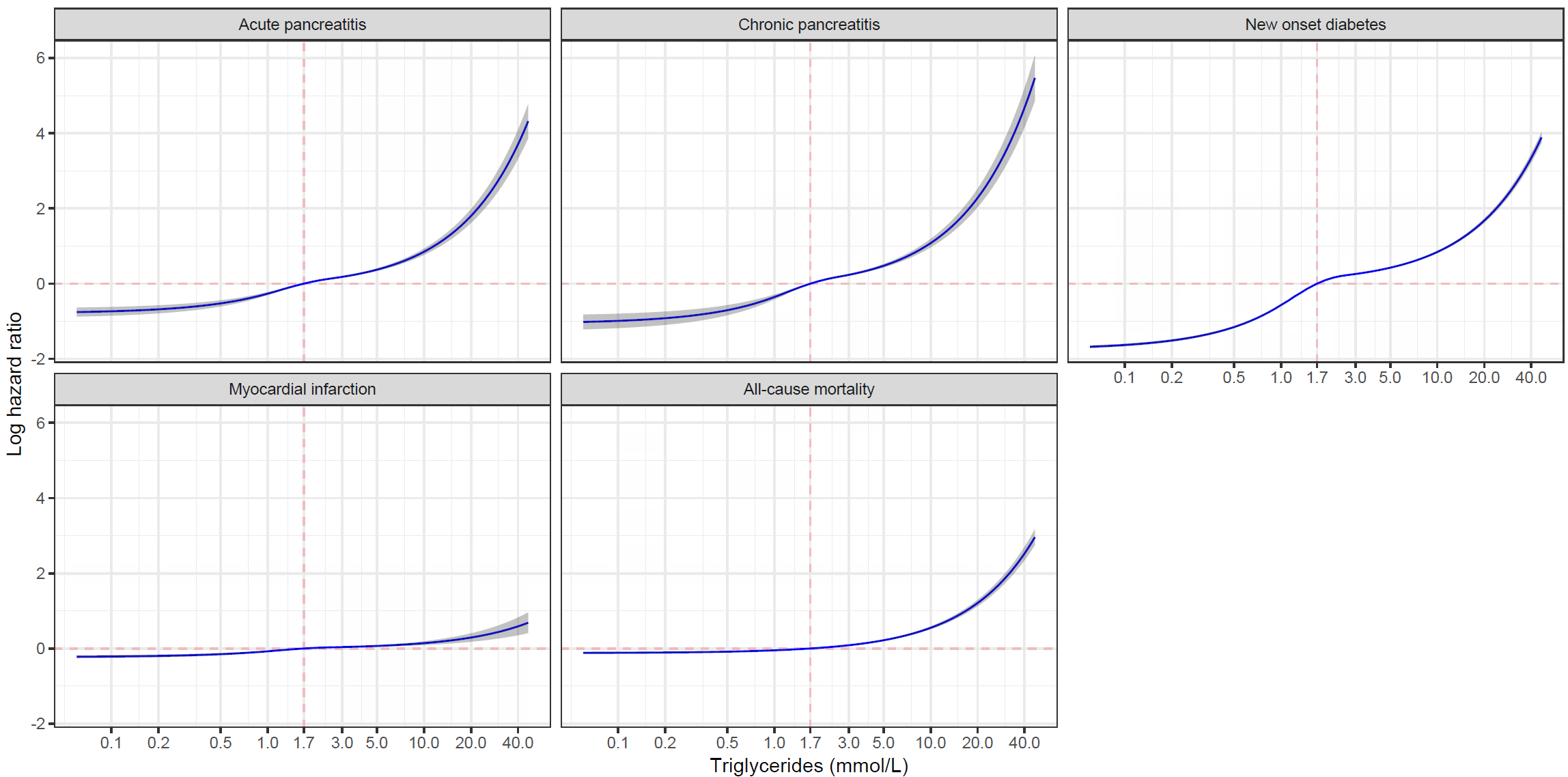
**

**Supplementary table 5: Hazard ratios and 95% confidence intervals from multivariable cox regression models using patients peak triglyceride record over 12 months**

| **TG vs. ≤1.7 (mmol/L)** | **Acute pancreatitis** | **Chronic pancreatitis** | **New onset diabetes** | **Myocardial infarction** | **All-cause mortality** |
| --- | --- | --- | --- | --- | --- |
| **(1.7, 4.5]** | 1.42 (1.17, 1.72) | 2.19 (1.68, 2.85) | 0.90 (0.85, 0.95) | 1.25 (1.19, 1.32) | 1.12 (1.07, 1.17) |
| **(4.5, 10]** | 1.32 (1.25, 1.39) | 1.42 (1.30, 1.55) | 1.74 (1.72, 1.76) | 1.06 (1.05, 1.08) | 1.07 (1.06, 1.09) |
| **(10, 20]** | 2.06 (1.83, 2.33) | 3.03 (2.53, 3.63) | 2.69 (2.61, 2.77) | 1.16 (1.11, 1.21) | 1.41 (1.37, 1.46) |
| **>20** | 4.28 (3.26, 5.62) | 5.76 (3.88, 8.53) | 3.21 (2.97, 3.46) | 1.04 (0.90, 1.19) | 1.93 (1.72, 2.17) |

**Supplementary table 6: Hazard ratios and 95% confidence intervals from multivariable cox regression models using complete-cases only (n=505,579 and n= 446,091 for the subset of diabetes-free patients)**

| **TG vs. ≤1.7 (mmol/L)** | **Acute pancreatitis** | **Chronic pancreatitis** | **New onset diabetes** | **Myocardial infarction** | **All-cause mortality** |
| --- | --- | --- | --- | --- | --- |
| **(1.7, 4.5]** | 1.27 (1.16, 1.39) | 1.42 (1.22, 1.64) | 1.75 (1.71, 1.79) | 1.04 (1.01, 1.07) | 1.05 (1.03, 1.07) |
| **(4.5, 10]** | 1.92 (1.53, 2.41) | 2.71 (1.98, 3.71) | 3.18 (3.02, 3.34) | 1.14 (1.05, 1.23) | 1.34 (1.25, 1.43) |
| **(10, 20]** | 4.27 (2.43, 7.50) | 4.16 (1.81, 9.58) | 5.69 (4.92, 6.58) | 0.86 (0.62, 1.19) | 2.29 (1.80, 2.91) |
| **>20** | 10.12 (4.05, 25.32) | 21.26 (7.48, 60.39) | 5.70 (4.07, 7.99) | 0.96 (0.45, 2.02) | 4.11 (2.38, 7.10) |
